# Fluxomic Analysis Reveals Central Carbon Metabolism Adaptation for Diazotroph *Azotobacter vinelandii* Ammonium Excretion

**DOI:** 10.1038/s41598-019-49717-6

**Published:** 2019-09-13

**Authors:** Chao Wu, Ryan A. Herold, Eric P. Knoshaug, Bo Wang, Wei Xiong, Lieve M. L. Laurens

**Affiliations:** Bioenergy Science and Technology Directorate, National Renewable Energy Laboratory (NREL), 15013, Denver West Parkway, Golden, CO 80401 USA

**Keywords:** Metabolomics, Computational models

## Abstract

Diazotrophic bacteria are an attractive biological alternative to synthetic nitrogen fertilizers due to their remarkable capacity to fix atmospheric nitrogen gas to ammonium via nitrogenase enzymes. However, how diazotrophic bacteria tailor central carbon catabolism to accommodate the energy requirement for nitrogenase activity is largely unknown. In this study, we used *Azotobacter vinelandii* DJ and an ammonium excreting mutant, AV3 (ΔNifL), to investigate central carbon metabolism fluxes and central cell bioenergetics in response to ammonium availability and nitrogenase activity. Enabled by the powerful and reliable methodology of ^13^C-metabolic flux analysis, we show that the respiratory TCA cycle is upregulated in association with increased nitrogenase activity and causes a monotonic decrease in specific growth rate. Whereas the activity of the glycolytic Entner–Doudoroff pathway is positively correlated with the cell growth rate. These new observations are formulated into a ^13^C-metabolic flux model which further improves the understanding and interpretation of intracellular bioenergetics. This analysis leads to the conclusion that, under aerobic conditions, respiratory TCA metabolism is responsible for the supply of additional ATP and reducing equivalents required for elevated nitrogenase activity. This study provides a quantitative relationship between central carbon and nitrogen metabolism in an aerobic diazotroph for the first time.

## Introduction

Reduced forms of nitrogen are required by every living organism and are used to make many important biological components including nucleic acids, proteins, pigments, energy carrier molecules, etc. In nature, a diverse group of bacteria known as diazotrophs have a remarkable capacity to fix atmospheric nitrogen to ammonium under ambient conditions. This capacity is catalyzed by the nitrogenase metalloenzyme complexed with accessory proteins for proper cofactor biosynthesis and can be exploited for biological fertilizer production. This process is accomplished on an industrial scale by the chemical process (Haber-Bosch) that requires elevated temperature, high pressure and special catalysts and is considered an energy intensive and major greenhouse gas emitting approach^1^. The ability of diazotrophs to use dinitrogen gas as the sole nitrogen source confers many ecological merits, but also incurs physiological constraints resulting from the high energy requirements of biological nitrogen fixation. The function of the nitrogenase enzyme requires not only reducing equivalents but also the supply of a minimum of 16 ATP per N_2_ fixed. Substantial energetic equivalents require a coordinated central carbon metabolism which uses a series of enzymatic pathways (*i.e*. the Embden-Meyerhof-Parnas (EMP) pathway, the Entner-Doudoroff (ED) pathway, pentose phosphate pathway, tricarboxylic acid (TCA) cycle etc.) to convert organic carbohydrates into metabolic precursors and release electrons and ATP. However, to date, there is little understanding of how diazotrophic bacteria quantitatively tailor central carbon catabolism to nitrogen availability and vice versa.

In addition to its substantial energy cost, biological nitrogen fixation is also highly oxygen sensitive. To protect the nitrogenase complex from damage, some aerobic diazotrophs have evolved the ability to form specialized cells that create a microanaerobic environment for nitrogen fixation to occur (*e.g*. heterocysts in nitrogen-fixing filamentous cyanobacteria). Alternatively, the non-compartmentalized diazotroph, *Azotobacter vinelandii*, is believed to have a different protection mechanism^2–4^ that exhibits high respiratory activity, especially when exposed to high oxygen concentrations. In addition, in environments where a high rate of respiration does not prevail (*e.g. A. vinelandii* grown in phosphate-limited nitrogen-free chemostat culture), cells produce extracellular alginate, a viscous polysaccharide that leads to the formation of a capsule surrounding the cell that prevents oxygen diffusion into the cell^5^. In order to adapt to different environmental conditions while accommodating nitrogenase activity and ensuring ammonium availability, intracellular carbon flux may need to be directed toward either respiratory metabolism or alginate biosynthesis.

*Azotobacter* is an attractive organism for biofertilizer applications because this species is capable of fixing atmospheric nitrogen under ambient conditions, is genetically tractable, and has been shown to excrete excess fixed ammonium^6–10^. For example, mutations in the nifL regulatory domain of the nitrogen fixation operon in *A. vinelandii* has yielded a mutant strain (AV3) that exhibits constant expression of nitrogenase, and thus increased ammonium production and excretion into the surrounding media^8^. However, this mutant exhibits metabolic mechanisms of nutrient balancing that could favor biomass production and may thus oppose the metabolic engineer’s goal of maximizing ammonia yield. Therefore, understanding how *A. vinelandii* coordinates the utilization of carbon with the rate of nitrogen fixation is critical for the successful reprogramming of this microbe’s metabolism. Therefore, we aim here to understand how ammonium excretion is adding a metabolic energetic burden to the cells’ metabolism. Using ^13^C-metabolic flux analysis, by coupling chromatography to mass spectrometry we are able to quantify the isotopomer patterns of metabolites throughout the metabolic network. This method has emerged as a powerful tool for generating the quantitative data needed to probe and model central carbon metabolism and is uniquely applicable to provide novel bioenergetic insights into carbon and nitrogen metabolism^11,12^.

The primary goal of this work is to understand quantitative carbon catabolism tailored to nitrogen availability and in particular in relation to mutations that yield high levels of nitrogen excretion. In this work, we present a quantitative fluxome analysis showing how central carbon metabolism is reprogramed in relation to nitrogen perturbation in diazotroph *A. vinelandii* DJ wild type (wt) and its ammonium-excreting strain, AV3 (ΔNifL)^8^. To our knowledge, this is the first study to quantify the relationship between central carbon and nitrogen metabolism in this model aerobic diazotroph.

## Results

### *A. vinelandii’s* growth correlates with ammonium availability and nitrogenase activity

**Figure 1:**
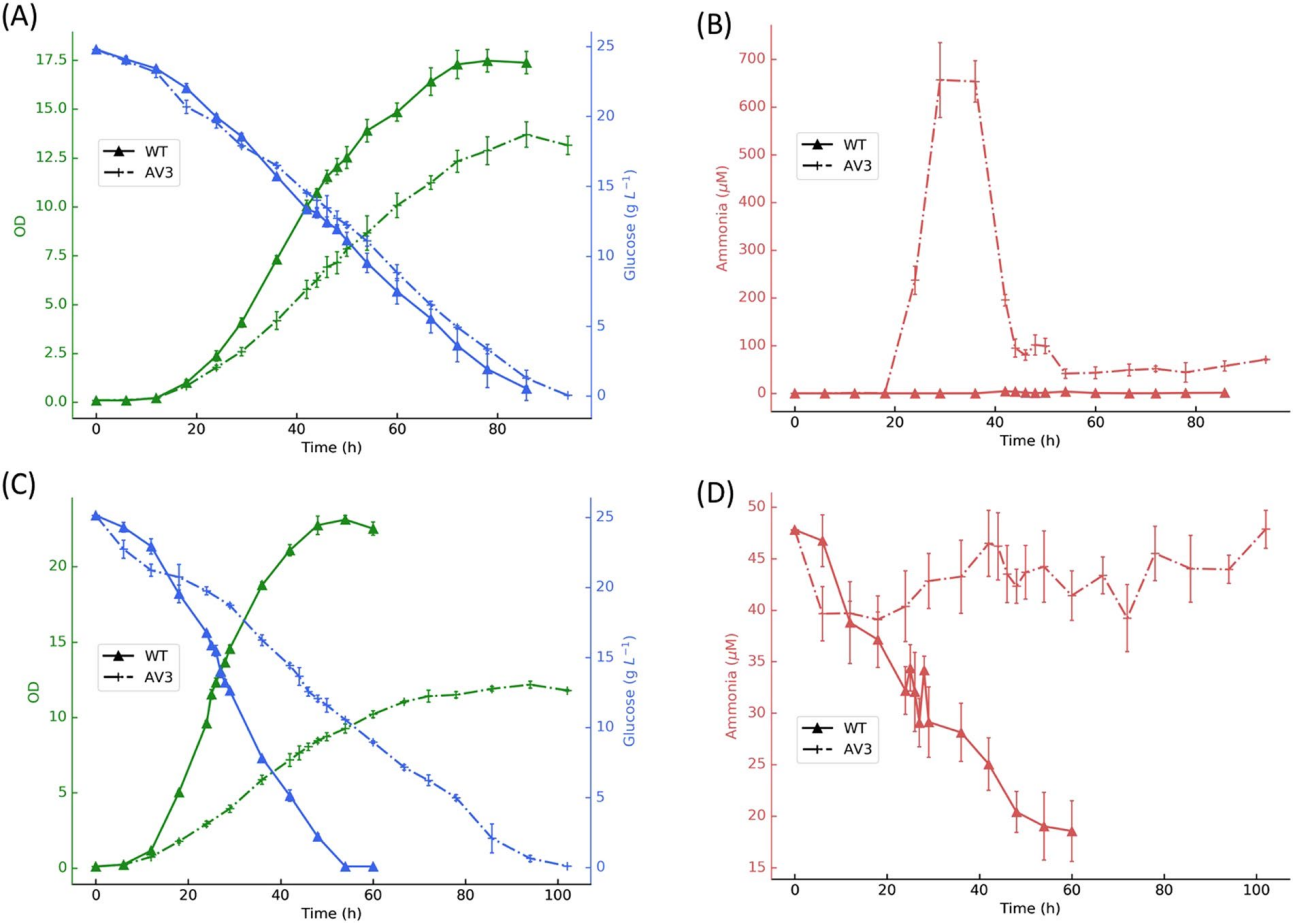
Growth curves of *A. vinelandii* wt and AV3 (**A**,**B**) without and (**C**,**D**) with 50 mM ammonium. OD (green), glucose (blue), and ammonium (red) were determined during cultivation of *A. vinelandii* wt (solid line with triangles) and AV3 (dashed line with crosses). Data points with error bars represent mean ± standard deviation of three replicates.

To investigate the influence of ammonium level on central carbon metabolism under different nitrogenase activity and environmental availability, we grew *A. vinelandii* wild type (DJ) and the ammonium excreting strain (AV3) with and without 50 mM ammonium (NH_4_Cl) added to the culture medium. The kinetics of cell growth, glucose consumption and ammonium levels in the media are shown in Fig. 1. Cell biomass was the predominant product and extracellular alginate was not detected in any of the conditions indicating that alginate-mediated nitrogenase protection is not the predominate strategy in the tested conditions (data not shown). Instead, we found a significant amount of poly 3-hydroxybutyrate (PHB) serving as a storage carbon polymer inside the cell (Table 1), and the PHB content in wt almost doubled compared with the AV3 mutant in media with and without ammonium suggesting a shortage of energy and reducing power in AV3 cells.

**Table 1:** Growth parameters of *A.vinelandii* wt and AV3 strain without and with additional ammonium.

		Maximum growth rate (h^−1^)	Biomass yield on glucose (g g^−1^)	Glucose uptake rate (mmol gDCW^−1^ h^−1^)	PHB content (%)	PHB formation rate (mmol gDCW^−1^ h^−1^)
without ammonium	wt	0.250 ± 0.020	0.180 ± 0.018	15.68 ± 0.65	57.0 ± 0.8	4.00 ± 0.49
	AV3	0.230 ± 0.005	0.095 ± 0.008	25.07 ± 1.45	31.6 ± 2.4	1.89 ± 0.54
with ammonium	wt	0.270 ± 0.004	0.208 ± 0.056	16.29 ± 3.48	56.1 ± 3.3	4.34 ± 0.22
	AV3	0.195 ± 0.001	0.071 ± 0.008	27.64 ± 2.69	33.6 ± 3.3	1.72 ± 0.15

Note. The maximum specific growth rate was calculated between 12 and 18 hrs. One mole of PHB is represented by 3HB. Alginate was not detected in tested conditions. Data are presented as mean ± SD of triplicate measurements.

In cultures grown without added ammonium (Fig. 1A), both the wt and AV3 strains grew diazotrophically. The AV3 strain excreted ammonium into the medium promptly within the first 24 hours, consistent with expectations resulting from increased nitrogenase expression. We observed that AV3 grew slower than wt strain in the exponential growth phase (maximum growth rate of 0.23 vs. 0.25, respectively) while incurring a higher glucose consumption rate (25.07 vs. 15.68 mmol gDCW^−1^ h^−1^, respectively). Interestingly, in linear and stationary growth phase the AV3 strain seemed to re-assimilate the ammonium it produced earlier, indicating that nitrogenase expression might be down-regulated by other unknown mechanisms, or the ammonium was consumed by spontaneous mutants (Nif^−^) that were unable to fix nitrogen.

For cultures grown with 50 mM added ammonium (Fig. 1B), AV3 maintained extracellular ammonium levels within 40–50 mM, while the wt consumed all available ammonium, showing the ability of the wt to use NifL to switch off nitrogenase expression when extracellular ammonium is present. Similar to the results in diazotrophic conditions, the higher expression of nitrogenase in AV3 caused it to grow much slower than the wt. For both wt and AV3, biomass formation was significantly slower under diazotrophic conditions compared to cultures with added ammonium. Overall, our physiological data indicate that when *A. vinelandii* is forced to fix nitrogen (in diazotrophic growth) due to the unavailability of ammonium, biomass formation and growth rate are significantly impeded. In addition, further increase in nitrogenase activity in the case of AV3, impedes the cells’ growth rate even further.

### ^13^C-Metabolic flux analysis by GC-MS of proteinogenic amino acids in *A. vinelandii* glucose batch culture

To investigate central carbon metabolism in relation to nitrogen status, we established a rapid and informative methodology for ^13^C-metabolic flux analysis (MFA). We developed ^13^C-MFA using isotope tracer and GC-MS to resolve the primary pathways in the carbon metabolism of *A. vinelandii*. For this purpose, we first validated growth on a minimal medium, modified DSMZ *Azotobacter* medium, to eliminate potential interference with labeled amino acids from non-labeled amino acids present in complex carbon nutrients (e.g., amino acids in yeast extract) that can be incorporated by the cells. Second, we found the fractional labeling (FL) of the GC-MS-analyzed proteinogenic amino acids to be generally consistent with that of the isotopic substrates when sampled from exponentially grown cultures (Supplemental Fig. 1). This ensures metabolic steady state conditions for isotopic labeling allowing quantitative flux estimation^11^. This labeling pattern is consistent with previously documented, and generally accepted report on the application of metabolic steady state or labeling steady state^11^. We performed GC-MS-based metabolic flux ratio analysis^13^ of shake-flask-grown *A. vinelandii* batch cultures on ^13^C-glucose at 30 °C in biological duplicates. Analysis of parallel cultures showed consistent results with only with minor derivations in flux ratios. Since the interpretation of ^13^C-labelling pattern in amino acids by MS is uniformly deduced by an identical biochemical network from experimental measurements, this consistency provides evidence for reliable quantification of metabolic flux ratios by this method.

### Glycolytic flux from the ED pathway corresponds to specific growth rate

**Figure 2:**
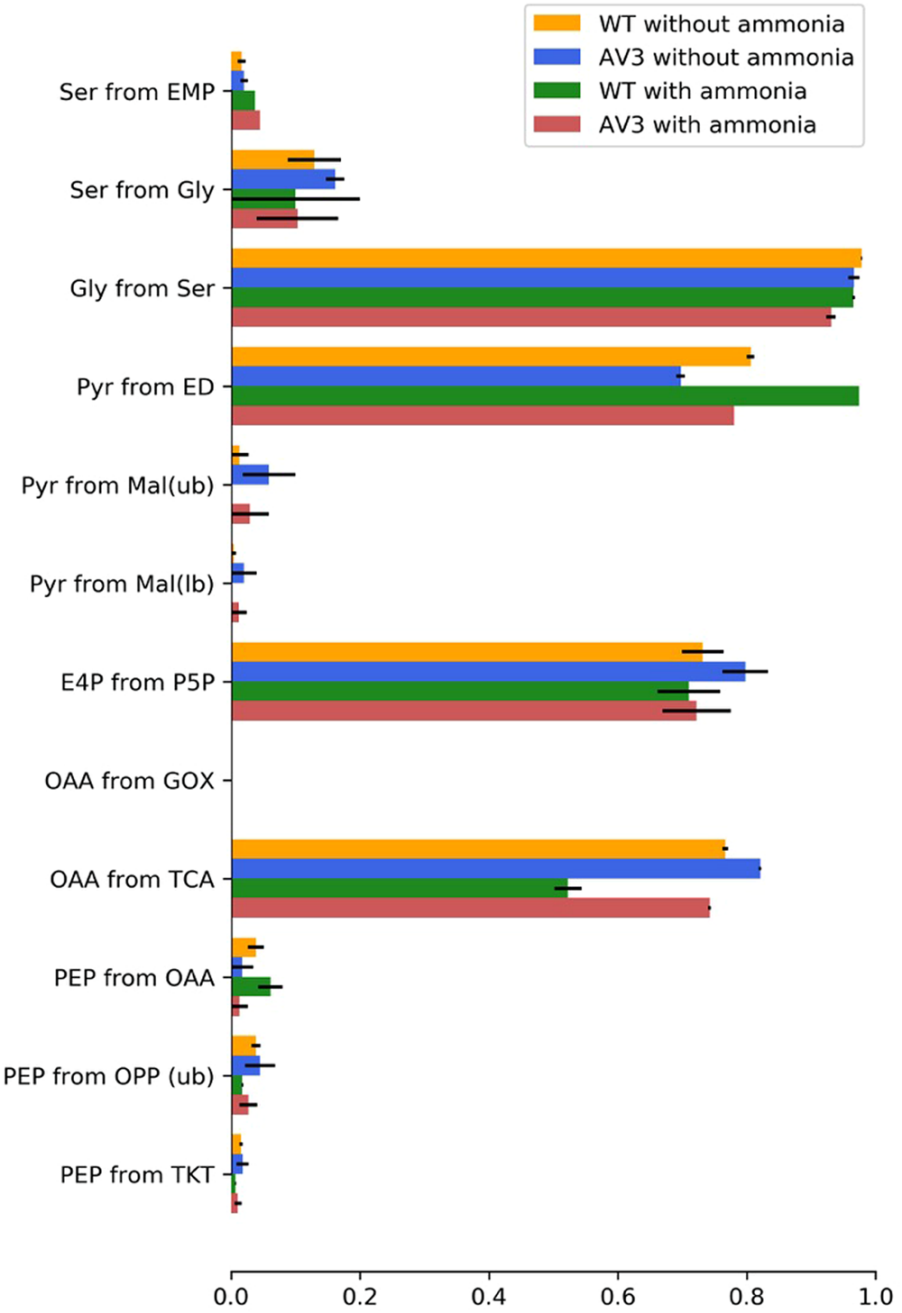
Metabolic flux ratios of intermediates in *A. vinelandii* wt and AV3 strain with and without ammonium. Ser from glycolysis and pyr from ED pathway are calculated using 100% 1-^13^C glucose. Other flux ratios are calculated from 20% U-^13^C glucose and 80% natural glucose. Data represent the mean of two replicates, and error was also estimated. Abbreviations: E4P, erythrose-4-phosphate; Gly, glycine; Mal, malate; OAA, oxaloacetate; OPP, oxidative pentose phosphate pathway; P5P, pentose-5-phosphate; PEP, phosphoenolpyruvate; Pyr, pyruvate; Ser,serine; ED, Entner-Doudoroff pathway; EMP, Embden-Meyerhof-Parnas pathway; GOX, glyoxylate shunt; TCA, tricarboxylic acid cycle; TKT, transketolase.

Flux ratio estimation from the 1-^13^C-glucose feeding experiment helped us to resolve the relative glycolytic flux through the EMP pathway, the ED pathway and the oxidative pentose phosphate (OPP) pathway. Although the complete gene sets for these pathways are found in *A. vinelandii’s* genome^14^, their metabolic contribution to glycolysis is not fully understood. Quantitative estimations of flux ratios through these pathways were based on the labeling patterns of serine and alanine as defined by Mass Distribution Vectors (MDV)^15^. If serine originates through the EMP pathway, half of the serine will be labeled at position C3 while the other half will be unlabeled (Materials and Methods, Supplemental Fig. 2). If serine exclusively originates from the oxidative PP pathway, none of it will be labeled because the ^13^C-labeled carbon at position C1 would be lost as CO_2_ in the oxidative reaction of the PP pathway (Supplemental Fig. 2). Serine derived from the ED pathway also yields unlabeled triose-3P molecules (Supplemental Fig. 2), while generating pyruvate molecules labeled at the C1 position, which is reflected in alanine as the product of transamination. We thus checked the MDV_ser1-3_ and MDV_ser1-2_ in all conditions. For example, cultures of *A. vinelandii* AV3 grown with added ammonium had MDV_ser1-3_ and MDV_ser1-2_ values of [0.9469, 0.0529, 0.0001, 0.0001]^T^ and [0.9784, 0.0215, 0.0001]^T^, respectively. These results suggest that serine is only minorly labeled at the position C3, consistent with non-negligible but relatively low activity of the EMP pathway (Fig. 2). We also checked the labeling pattern of alanine by MDV_ala1-3_ and MDV_ala2-3_. in the wt strain with added ammonium and found [0.5005, 0.4887, 0.0108, 0.0001]^T^ and [0.9738, 0.0239, 0.0023]^T^, respectively. In every scenario we found that a significant fraction of alanine was labeled at the carbonyl carbon (position C1), strongly supporting that the ED pathway is the dominant pathway for glycolysis. Interestingly, we found the ED pathway flux corelates with the specific growth rate as the fastest growing cells carried the strongest ED pathway, and vice versa (Fig. 2). This result reveals not only the absolute velocity of glucose utilization but also that proportional glycolytic flux through the ED pathway serves as key parameters affecting cell growth rate. In addition, by subtracting the constitutive fluxes from the ED and EMP pathway, we can also interpret that the oxidative part of the pentose phosphate pathway is relatively low in *A. vinelandii*.

### Cyclic flux of the TCA cycle negatively correlates with the specific growth rate

**Figure 3:**
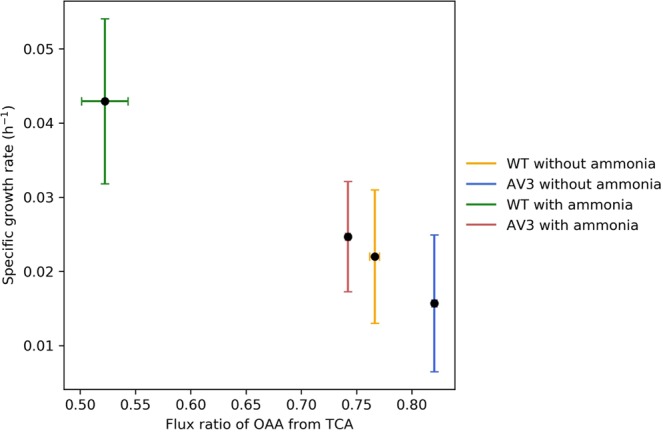
Negative correlation between TCA cycle flux and calculated specific growth rate of *A. vinelandii* in different environment and genetic background. Data points with vertical error bars represent mean ± standard deviation of three replicates. Data points with horizonal error bars represent mean ± standard deviation of two replicates.

We further focused on the respiratory flux from the TCA cycle. This flux can be reflected by the proportion of oxaloacetate (OAA) synthesized from either the TCA cycle or amphibolic pathway. The increased fraction of OAA derived through the TCA cycle in the AV3 culture without added ammonium indicates significantly increased flux through the respiratory TCA cycle. This data aligns with the notion that enhanced nitrogenase activity requires highly efficient respiratory metabolism to produce additional ATP. Interestingly, we also found that the relative respiratory activity of the TCA cycle increased as the growth rate decreased in cultures for different ammonium scenarios, with the most extreme negative relation in the case of diazotrophic growth of the AV3 mutant (Fig. 3). In addition, TCA cycle activity was found to be a decreasing function of biomass yield on glucose, consistent with its role in competitively oxidizing acetyl CoA, the primary biomass building block, to CO_2_. In addition to the OAA originating from the cyclic TCA cycle, the remaining OAA is synthesized from phosphoenolpyruvate (PEP) proportionally by PEP carboxylase as an amphibolic reaction. In comparison, the backward fluxes such as “PEP from OAA”, “Pyr from Mal (lb and ub)” are negligible.

### Global flux profiling demonstrates bioenergy and redox production from central carbon metabolism

*In vivo* biochemical reaction rates are among the most important metabolic phenotypes to understand. In order to obtain quantitative insight into the global carbon fluxes in *A. vinelandii*, we developed a metabolic model that describes isotope labeling of metabolites upon the addition of 20% universally labeled ^13^C-glucose and 80% unlabeled glucose (see Materials and Methods for modeling details). The biochemical reactions and biomass equation adopted in our model come from Andres’s work^16^ and are listed in Supplemental Table 1, which is based on genomic information and involves validated pathways that have been identified by our isotope tracer experiments as described above. Specifically, the principal metabolic network includes the ED pathway, the EMP pathway, the OPP pathway, anaplerotic reactions, and the TCA cycle, all of which have been verified by isotope tracer experiments based on this work. In addition, we included the glyoxylate shunt into the central carbon metabolism which is present in the *A. vinelandii* genome but showed marginal activity according to flux ratio analysis as presented in Fig. 2.

**Figure 4:**
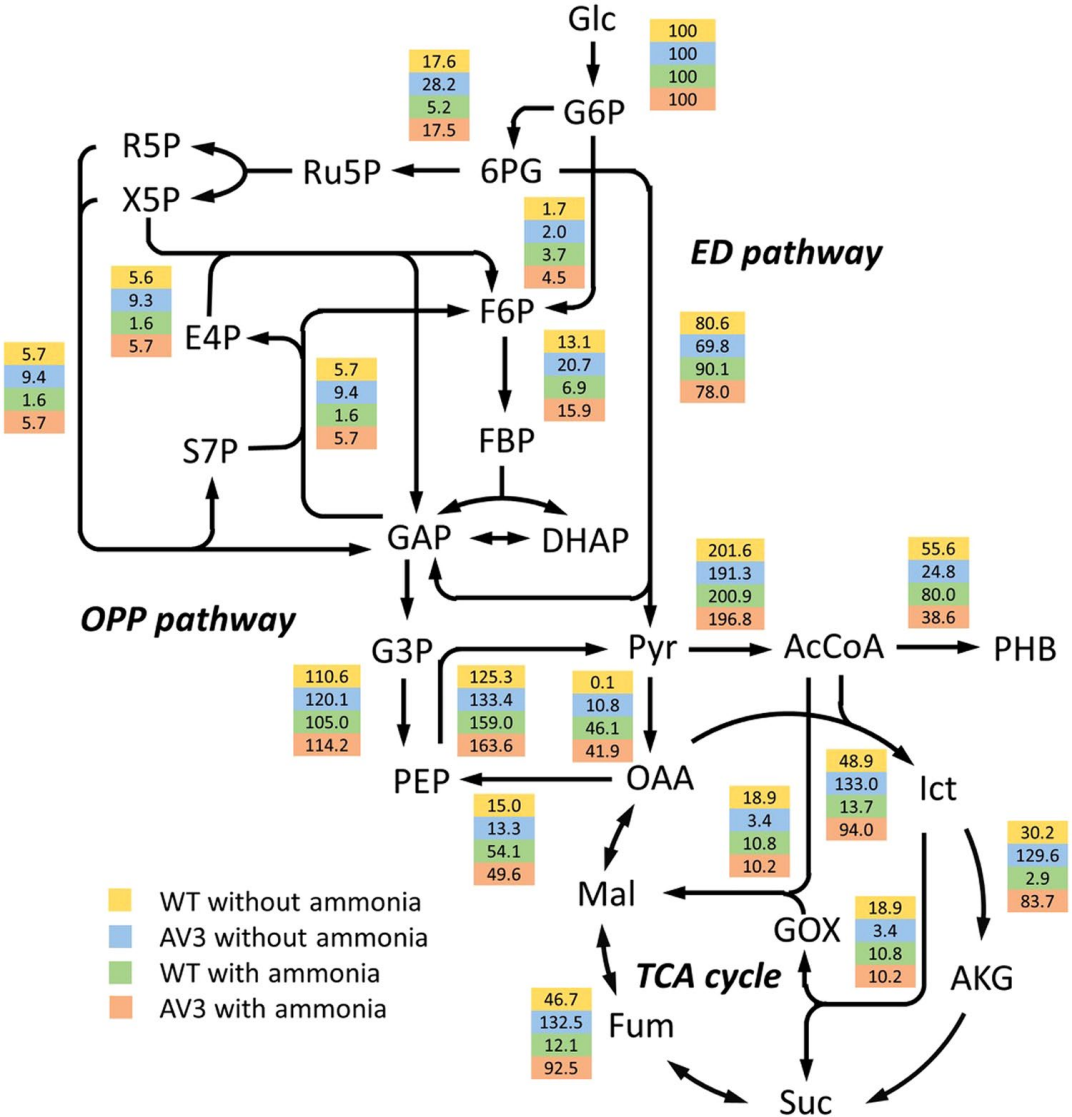
Metabolic flux maps of *A. vinelandii* wt and AV3 with and without ammonium. Metabolic fluxes are best estimated using the MDVs of proteinogenic amino acids labeled from a mixture of 20% U-^13^C glucose and 80% unlabeled glucose. Constraints provided by 100% 1-^13^C glucose as well as growth parameters. Values of metabolic flux are normalized to a glucose uptake rate of 100. Abbreviations: 6PG, 6-phosphogluconate; AcCoA, acetyl coenzyme A; AKG, a-ketoglutarate; Ict, isocitrate; DHAP, dihydroxyacetone phosphate; E4P, erythrose-4-phosphate; F6P, fructose-6-phosphate; FBP, fructose-1,6-bisphosphate; Fum, fumarate; G3P, 3-phosphoglycerate; GAP, glyceraldehyde-3-phosphate; Glc, glucose; PHB, polyhydroxybutyrate; R5P, ribose-5-phosphate; Ru5P, ribulose-5-phosphate; S7P, sedoheptulose-7-phosphate; Suc, succinate; X5P, xylulose-5-phosphate.

The estimated fluxes from the model fit are presented in Supplemental Table 2 and corresponding fitting results are shown in Supplemental Fig. 3. The fluxomic model fits the observed data, and most of the identified fluxes have modest confidence intervals, indicating that they are reliable to experimental measurements. Figure 4 shows a map of the estimated flux values in central carbon metabolism. The results show that glycolytic flux predominates through the ED pathway and is directed primarily toward pyruvate formation. Pyruvate is directed to acetyl CoA by pyruvate dehydrogenase and acetyl CoA is channeled into the TCA cycle, biomass generation, or PHB synthesis proportionally, depending on the ammonium availability and nitrogenase activity. Other apparent fluxes include oxaloacetate production via PEP carboxylase, and pentose-phosphate production from glycolytic intermediates. The computational results presented herein demonstrate a characteristic fluxomic structure in *A. vinelandii*.

**Figure 5:**
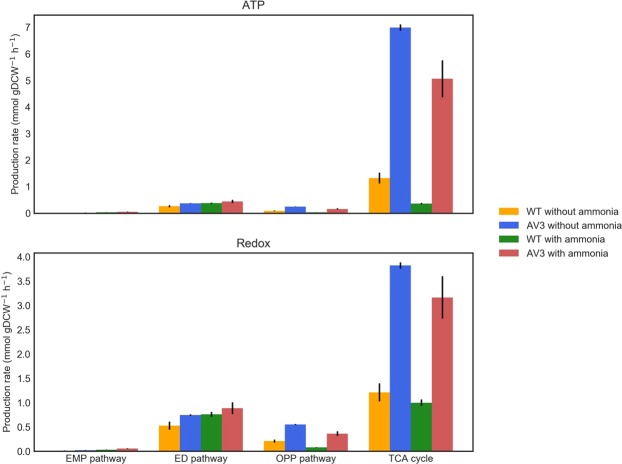
Energy and reducing equivalent generation in *A. vinelandii* wt and AV3 with and without ammonium. ATP and redox production rates were obtained from estimated fluxes and stoichiometry of corresponding reactions. Note that NADH and FADH2 generated in the TCA cycle are consumed in the electron transport chain to produce ATP under an assumed P/O ratio of 2.5 and 1.5 respectively.

The global fluxomics datasets enable us to estimate the bioenergy and redox production from the stoichiometry of the metabolic network (Fig. 5). We thus compared the ATP and reducing equivalents yields from the primary carbon pathways (the EMP pathway, the ED pathway, the OPP pathway and the TCA cycle) in all tested conditions and strains. We found that the TCA cycle is the dominant bioenergy generator accounting for over 45% production of of ATP and reducing equivalents. Strikingly, these productions dramatically increase with enhanced nitrogenase activity in the AV3 mutant. In cultures with and without added ammonium, the AV3 strain produced substantially more ATP (89–92%) and redox cofactors (71–74%) than the wt (45–78% and 54–62%, respectively) from the TCA cycle. Our analysis supports the notion that the TCA cycle output satisfies the majority of the energetic requirements for biological nitrogen fixation (Fig. 5). We also compared the ATP and NADPH requirement for nitrogen fixation based on our calculated fluxes and measured ammonium (Supplemental Fig. 4), which is compatible with the estimated the bioenergy and redox production. It is worth noting that even under exogenous ammonium there’s still an energy consumption tied to nitrogen fixation in the mutant, which is consistent with a sustained extracellular ammonium production.

## Discussion

Using ^13^C-tracer studies, we developed a fluxomic model to formulate the quantitative relationship between central carbon fluxes and nitrogen metabolism in *A. vinelandii* and elucidated the integrated responses of metabolic networks to nitrogen perturbation. Using this fluxomic model framework we were able to probe the bioenergetic basis of a diazotrophic phenotype in the ammonium-excreting AV3 mutant, which allows further refinement of the model to guide strategies toward metabolic engineering goals.

Our study sheds light on the glycolytic strategy used by diazotroph *A. vinelandii*, which carries complete gene sets for two critical glycolytic options, the EMP and ED pathways. This work shows that *A. vinelandii* predominantly utilizes the ED pathway, while the EMP pathway has very low activity. Similar glycolytic flux partitioning is also observed in *Pseudomonas*^17,18^, a genus phylogenetically close to *Azotobacter*, in which the ED pathway accounts for over 90% of utilized glucose^19,20^. These two glycolytic pathways vary in reaction schemes and in how much ATP they produce for each glucose molecule metabolized^21^. Given the substantial energetic requirements for diazotrophic growth, why *A. vinelandii* prioritizes the ED pathway for glycolysis represents an intriguing open question. Compared with the EMP pathway, the most energetically efficient glycolytic pathway, the ED pathway oxidizes phosphorylated glucose to 2-keto-3-deoxy-6-phosphogluconate (KDPG), which is cleaved into one pyruvate and one glyceraldehyde-3-phosphate (G3P). Only the latter product supports substrate-level phosphorylation and can be used to produce ATP through subsequent glycolysis. Despite reduced ATP production, the ED pathway contains more exergonic reactions^22^ and is less thermodynamically constrained. This feature leads to tremendous benefit in enzyme kinetics as a stronger thermodynamic driving force will result in forward reactions being favored, which accordingly requires reduced amounts of catalytic enzymes to generate identical net flux. Indeed, the ED pathway could reduce by several-fold the enzymatic protein synthesis burden but still achieve the same glucose conversion rate as the EMP pathway^21^. It should be noted that in diazotrophic growth, the cost for protein synthesis can be considerable as it is largely based on the energetically expensive nitrogen-fixing process. From this perspective, natural selection of the ED pathway represents an energy balance consideration geared towards higher conversion rates of carbon substrates per N_2_ fixed and constrained by protein synthesis demands. The trade-off of this strategy is the decreased production of ATP from glycolysis, which must be replenished through another oxidative pathway as discussed below. Interestingly, our data also showed that the flux ratio of the ED pathway is positively correlated with the cell growth rate constraints by either ammonium availability or nitrogenase activity. The biological mechanism behind this correlation is still unknown. One hypothesis is that the activities of the ED pathway enzymes could be regulated by altered intracellular ammonium levels regardless of transcriptomic, proteomic, or metabolomic levels. Further research in systems biology is required to address this question comprehensively.

In addition, our ^13^C-fluxomic study also provides new evidence to better understand the role of respiratory metabolism in diazotrophs. Respiratory metabolism was proposed to protect the active nitrogenase against oxygen because the respiratory electron transport system bound to the peripheral cytoplasmic membrane can scavenge O_2_ and prevent the diffusion of O_2_ into the cells. This mechanism is believed to explain how nitrogenase can function when cells grow diazotrophically in the presence of O_2_. Although the respiration-protection hypothesis is widely accepted, it was recently challenged when it was reported that *A. vinelandii* had almost constant respiration rates and nitrogenase activity at O_2_ concentrations ranging from 30 to 100% air saturation^23,24^. These results hardly support the concept of respiratory protection. As a complementary hypothesis, Oelze suggested that nitrogenase protection also depends on the maintenance of a sufficiently low redox state^25^. This hypothesis assumes that oxidation of the substrate can warrant the proper supply of reducing equivalents and ATP to maintain nitrogenase in a reduced state. Indeed, our data strongly supports this hypothesis in that the increased fluxes through respiratory TCA cycle and OPP pathway positively correlated with enhanced nitrogenase activity. As shown in Fig. 2, the AV3 mutant exhibited remarkably higher metabolic activity in TCA cycle than wt strain in both media with and without supplemented ammonium, which suggests that sufficient ATP and reducing equivalents are produced when there is strong N_2_ fixation activity. It also supports the notion that respiratory protection arises from stringently coupled respiratory electron transport with the TCA metabolism. Although the way in which diazotrophs increase TCA cycle flux in response to enhanced nitrogenase expression is still a mystery, knowledge of this correlation could lead to lead to new strategies to maximize nitrogenase activity for engineering increased ammonium release by alleviating apparent metabolic bottlenecks. One solution could be the use of alternative carbon substrates (e.g. citrate and acetate) that can increase the activity of the TCA cycle locally, and accordingly increase the supply of energy and reducing equivalents. Previous studies on diazotrophically growing *A. vinelandii* showed that growth rates obtained on acetate achieved 0.35 h^−1^, higher than those grown on glucose (0.15 h^−1^)^26^. Therefore, addition of direct precursors of TCA cycle will not only supply ATP and reducing power for the highly active nitrogen fixation in the AV3 mutant, but also compensate for the carbon loss in TCA cycle which promises an enhanced biomass accumulation. It is noteworthy that the AV3 mutant showed a higher metabolic flux through the OPP pathway than wt in different ammonium availabilities which also supports the hypothesis in that extra reducing equivalents produced from an enhanced OPP pathway could be used to stabilize the enhanced nitrogenase activity in an AV3 mutant background. Overall, by deciphering the glucose metabolism of *A. vinelandii*, this work exemplifies novel insights into a general strategy that diazotrophs adopt for oxidizing carbon substrates under different conditions. It is our expectation that this quantitative knowledge of central carbon metabolism in terms of environmentally or genetically altered N-status can guide the reprogramming of diazotrophic metabolism geared toward increased technical feasibility of biologically produced ammonium fertilizers for industrial applications.

## Materials and Methods

### Strains and growth conditions

*Azotobacter vinelandii* DJ (wt) and AV3 (ΔNifL) strains were kindly provided by Dr. Leonardo Curatti^27^ and were maintained on 1.5% agar modified DSMZ (Leibniz Institute DSMZ-German Collection of Microorganisms and Cell Cultures) *Azotobacter* medium plates without added nitrogen. Our modified version of DSMZ *Azotobacter* medium consisted of 0.9 g L^−1^ K_2_HPO_4_, 0.1 g L^−1^ KH_2_PO_4_, 0.1 g L^−1^ MgSO_4_·7H_2_O, 0.1 g L^−1^ CaCl_2_·2H_2_O, 5 mg L^−1^ Na_2_MoO_4_·2H_2_O, 20 mg L^−1^ FeSO_4_·7H_2_O, 20 g L^−1^ glucose, 50 mM MOPS buffer, and was adjusted with HCl to a starting pH of 7.6. For cultures grown with added ammonium, NH_4_Cl was added to a final concentration of 50 mM prior to adjusting the pH. The MOPS buffer was included to prevent a drop in pH that truncated cell growth in cultures with added ammonium as has been previously reported^28^. Media was filter sterilized, and cultures were grown in unbaffled shake flasks at 30 °C and 225 rpm. Larger cultures of 250 mL in 1 L shake flasks were grown in triplicate to measure multiple metabolites during cell growth. U-^13^C and 1-^13^C glucose (Cambridge Isotope Laboratories, Tewksbury, MA, USA) labeled cultures consisted of 12.5 mL of culture in 50 mL shake flasks and were grown in duplicate. Cell growth was monitored by measuring optical density at 600 nm using a spectrophotometer (Beckman Coulter, Brea, CA, USA). Dry cell weight measurements were determined by centrifugation of a known volume of culture and subsequent lyophilization and recording of the pellet weight.

### Residual glucose quantification

Glucose was analyzed using a method slightly modified from literature^29^. Culture supernatants were filtered through 0.22 µm filters before being loaded on an Agilent 1100 series HPLC equipped with a refractive index detector (RID) (Agilent, Santa Clara, CA, USA) and an Aminex HPX-87H anion-exchange (Bio Rad Laboratories, Hercules, CA, USA). The mobile phase consisted of 4 mM H_2_SO_4_ at a constant flow rate of 0.6 ml min^−1^. The column temperature was maintained at 55 °C and the optical unit temperature for RID was held at 45 °C.

### Ammonium quantification

Ammonium was quantified using an assay kit (Abcam, Cambridge, UK) based on a modified version of the Berthelot method^30^. After incubating with reagent1 and reagent2 at 37 °C for 30 min, samples were analyzed with a microplate reader (Tecan, Männedorf, Switzerland).

### PHB quantification

Intracellular PHB was quantified using a modified esterification method^31^. Freeze-dried cells (about 3 mg) were weighed and added to 500 μL butanolysis solution (consisting of 90% (w/v) 1-butanol, 10% concentrated HCl and 2 mg mL^−1^ Benzoic acid as internal standard). The samples were incubated at 110 °C for 3 hrs followed by the addition of 750 μL Milli-Q water and vigorous voretxing for phase separation. An Agilent 7890B GC equipped with an Agilent HP-5 GC column was used to quantify the butyl ester of 3-hydroxybutyrate in the upper organic phase. Samples were analyzed at split ratio of 1:20 and overall flow rate of 1 mL min^−1^ with the following temperature profile: 90 °C, hold for 2 min; linear temperature gradient of 30 °C min^−1^ from 90 to 300 °C; 250 °C, hold for 2 min.

### Alginate quantification

Alginate was determined according to a polyhexamethylenebiguanidinium chloride (PHMBH^+^Cl^−^) method^32^. Briefly, culture supernatants were added to a PHMBH^+^Cl^−^ solution (purchased from BOC Sciences) (0.3% (w/v) in 1% sodium acetate) at volume ratio of 1:2 to form an alginate-PHMBH^+^ precipitate. After centrifugation at 17000 g for 5 min, absorbance of the supernatant with residual PHMBH^+^Cl^−^ was measured at 235 nm using NanoDrop One (ThermoFisher Scientific, Wilmington, DE, USA).

### GC-MS measurement for labeled amino acids

Sample preparation for GC-MS measurement of labeled amino acids followed the standard protocol^15^. The cells (OD_600_ * culture volume ≥5) were harvested at three time points (42, 46 and 50 hr for wt and AV3 without added ammonium, and AV3 with added ammonium; 25, 27 and 29 hr for wt with added ammonium) by centrifugation at 13000 g for 5 min. The specific growth rates (µ) calculated at these time points and used for the subsequent metabolic flux analysis was as follows: 0.022 ± 0.009, 0.016 ± 0.009, 0.043 ± 0.011, 0.025 ± 0.007 for wt and AV3 without added ammonium, and wt and AV3 with added ammonium, respectively. Cell pellets were washed with Milli-Q water and resuspended in 500 uL of 6 M HCl followed by incubation at 105 °C for 16 hrs. The hydrolysate was evaporated down under nitrogen gas flow at 40 °C, which can either be stored at −20 °C or used immediately for derivatization. Derivatization was carried out at 85 °C for 1 hr in a solution containing 80 uL pyridine and 20 uL N-methyl-N-(t-butyldimethylsilyl)-trifluoroacetamide. The derivatized sample was analyzed by GC-MS. The GC-MS parameters used were the same as previously described^33^.

### Calculations of metabolic flux ratios

Calculation of metabolic flux ratios was based on mass isotopomer distribution vector (MDV) of amino acid fragments as^15^:

$$MD{V}_{AA}=[\begin{array}{c}{m}_{0}\\ {m}_{1}\\ \ldots \\ {m}_{n}\end{array}]\,\,{\rm{with}}\,\mathop{\sum }\limits_{i=0}^{n}{m}_{i}=1$$

where m_i_ represents the fraction of the mass isotopomer with i carbon atoms labeled, and n is the number of carbons in the fragment. MDV_AA_ was then corrected for natural abundance of non-carbon atoms in fragment and carbon atom in the derivatization reagent. To prove that the cells were in a metabolic steady state, fractional labeling (FL) was calculated for U-^13^C glucose fed cells as^15^:

$$FL=\frac{\mathop{\sum }\limits_{i=0}^{n}i\cdot {m}_{i}}{n}$$

Mass isotopomer distribution of the precursors of amino acids (MDV_M_) can be further estimated via a least square method due to the convolution reaction of amino acid biosynthesis^15^, and flux ratios at metabolic nodes were calculated as least square solutions. In this study, flux ratios of serine from glycine, glycine from serine, pyruvate from malate (upper and lower bound), erythrose-4-phosphate from pentose-5-phosphate, oxaloacetate from the glyoxylate shunt, oxaloacetate from the TCA cycle, phosphoenolpyruvate from oxaloacetate, phosphoenolpyruvate from the PP pathway and phosphoenolpyruvate from transketolase were calculated according to the principles proposed by Fischer *et al*.^34^. Additionally, the flux ratios of serine from glycolysis, and pyruvate from the ED pathway were obtained using 1-^13^C glucose. If all G3P molecules originate through the EMP pathway, half of the resulting serine will be unlabeled and the other half will be labeled at position C3, while all serine molecules will be unlabeled if carbon flows through the ED pathway or the OPP pathway (Supplemental Fig. 2). Therefore, the flux ratio of serine from glycolysis can be solved according to:

$$\begin{array}{rcl}MD{V}_{Ser1-3} & = & {f}_{Ser\_from\_EMP}\cdot (0.5MD{V}_{3C\_from\_Glc}+0.5MD{V}_{unlabeled\_3C})\\  &  & +\,(1-{f}_{Ser\_from\_EMP})\cdot MD{V}_{unlabeled\_3C}\end{array}$$

Similarly, if all pyruvate originates from the ED pathway, alanine will be 50% unlabeled and 50% labeled at position C1 (Supplemental Fig. 2). Accordingly, flux ratio of pyruvate from ED pathway can be solved from:

$$\begin{array}{rcl}MD{V}_{Pyr1-3} & = & {f}_{Pyr\_from\_ED}\cdot (0.5MD{V}_{3C\_from\_glc}+0.5MD{V}_{unlabeled\_3C})\\  &  & +\,(1-{f}_{Pyr\_from\_ED})\cdot MD{V}_{PEP1-3}\end{array}$$

All calculations were implemented with Python3.6.

### Metabolic flux analysis

Primary metabolic network of *A. vinelandii* was reconstructed according to García *et al*.^16^ and provided in Supplemental Table 1. Functions between free metabolic fluxes and intermediate MDVs were established according to mass balance from reaction stoichiometry and isotopomer balance from atom transition using an elementary metabolic unit (EMU) method^35^. Determined MDVs and measured growth parameters were used as inputs for the following least-squares minimization problem^36^:

$$\begin{array}{ccc}min\,obj & = & \mathop{\sum }\limits_{i=1}^{m}(MD{V}_{sim,i}(u)-MD{V}_{exp,i})\cdot {\sum }_{exp,i}^{-1}\cdot {(MD{V}_{sim,i}(u)-MD{V}_{exp,i})}^{T}\\  &  & +\,\mathop{\sum }\limits_{i=1}^{n}\frac{{({v}_{sim,i}(u)-{v}_{exp,i})}^{2}}{va{r}_{exp,i}}\\ s.t.\,N\cdot u\ge 0\end{array}$$

where u is the vector of free fluxes; MDV_sim_ and v_sim_ are simulated mass isotopomer distribution and flux as a function of u, while MDV_exp_ and v_exp_ are experimental data; ∑_exp_ is square matrix with measurement variances of MDV elements on the diagonal; var_exp_ is the variance of determined reaction rate; N is the null space of stoichiometry matrix of mass balance. The problem can be solved through a gradient-based optimization algorithm. For flux estimation, the reaction rate of alginate formation was set to zero since alginate was not detected in any conditions. The flux between malate and pyruvate was also set to zero because the measured flux ratio of pyruvate from malate is negligible. The optimization was repeated 10 times for each condition to obtain a global solution, and 95% confidence intervals were calculated using a local linearization method, which is symmetric^36,37^. This method decreases computational burden compared to parameter continuation or Monte Carlo methods. All computation was implemented with Python3.6.

## Supplementary information

Fluxomics analysis of Azotobacter vinelandii supplemental information
