# Author Correction: Fluxomic Analysis Reveals Central Carbon Metabolism Adaptation for Diazotroph *Azotobacter vinelandii* Ammonium Excretion

**DOI:** 10.1038/s41598-019-54633-w

**Published:** 2019-11-26

**Authors:** Chao Wu, Ryan A. Herold, Eric P. Knoshaug, Bo Wang, Wei Xiong, Lieve M. L. Laurens

**Affiliations:** Bioenergy Science and Technology Directorate, National Renewable Energy Laboratory (NREL), 15013, Denver West Parkway, Golden, CO 80401 USA

Correction to: *Scientific Reports* 10.1038/s41598-019-49717-6, published online 13 September 2019

In Figure 1D, the y-axis is incorrectly labelled as ‘*µ*M’, it should read ‘mM’. The correct Figure 1D appears below.

**Figure 1:**
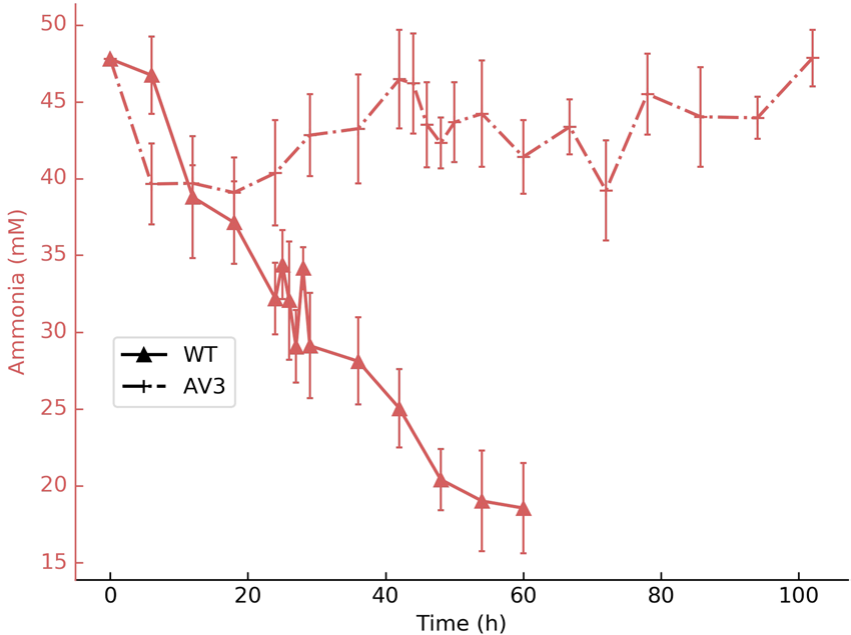
.

