# Supplementary material for: Fluxomic Analysis Reveals Central Carbon Metabolism Adaptation for Diazotroph Azotobacter vinelandii Ammonium Excretion
Source: Sci Rep. 2019 Sep 13;9:13209. doi: 10.1038/s41598-019-49717-6 (PMC6744558; doi:10.1038/s41598-019-49717-6)
Supplement: Supplementary file 1 — Fluxomics analysis of Azotobacter vinelandii supplemental information [file 41598_2019_49717_MOESM1_ESM.docx]

**Fluxomic Analysis Reveals Central Carbon Metabolism Adaptation for Diazotroph *Azotobacter vinelandii* Ammonium Excretion**

Chao Wu^1,§^, Ryan A. Herold^1,§^, Eric P. Knoshaug^1^, Bo Wang^1^, Wei Xiong^1,^*, Lieve ML. Laurens^1,^*

^1^ Bioenergy Science and Technology Directorate, National Renewable Energy Laboratory (NREL), 15013 Denver West Parkway, Golden, CO 80401, USA

^§^ Authors contributed equally to this work.

* Corresponding authors

Tel.: +1-303-384-7965; Fax: +1-303-384-7836; E-mail: [wei.xiong@nrel.gov](file:///C:\Users\wxiong\Desktop\NREL%20work\Chao%20Wu\Azotobacter%20manuscript\20181102\wei.xiong@nrel.gov)

Tel.: +1-303-384-6196; Fax: +1-303-384-6196; E-mail: [lieve.laurens@nrel.gov](mailto:lieve.laurens@nrel.gov)

**Supplementary Information**

**Supplemental Table 1**. Metabolic biochemical reactions and atomic transitions used for metabolic flux estimations in *A.vinelandii*.

| Reaction ID | Reaction with atom transition |
| --- | --- |
| v1 | Glc(abcdef) => G6P(abcdef) |
| v2 | G6P(abcdef) => F6P(abcdef) |
| v3 | F6P(abcdef) <=> FBP(abcdef) |
| v4 | FBP(abcdef) <=> DHAP(cba) + GAP(def) |
| v5 | DHAP(abc) <=> GAP(abc) |
| v6 | GAP(abc) <=> G3P(abc) |
| v7 | G3P(abc) <=> PEP(abc) |
| v8 | PEP(abc) => Pyr(abc) |
| v9 | G6P(abcdef) => KDPG(abcdef) |
| v10 | KDPG(abcdef) => Pyr(abc) + GAP(def) |
| v11 | G6P(abcdef) => Ru5P(bcdef)+CO2(a) |
| v12 | Ru5P(abcde) <=> X5P (abcde) |
| v13 | Ru5P(abcde) <=> R5P(abcde) |
| v14 | X5P(abcde) <=> TK(ab) + GAP(cde) |
| v15 | F6P(abcdef) <=> TK(ab) + E4P(cdef) |
| v16 | S7P(abcdefg) <=> TK(ab) + R5P(cdefg) |
| v17 | F6P(abcdef) <=> TA(abc) + GAP(def) |
| v18 | S7P(abcdefg) <=> TA(abc) + E4P(defg) |
| v19 | OAA(abcd) <=> PEP(abc) + CO2(d) |
| v20 | Pyr(abc) + CO2(d) => OAA(abcd) |
| v21 | Pyr(abc) => AcCoA(bc) + CO2(a) |
| v22 | OAA(abcd) + AcCoA(ef) => Cit(dcbfea) |
| v23 | Cit(abcdef) <=> Ict(abcdef) |
| v24 | Ict(abcdef) => AKG(abcde )+ CO2(f) |
| v25 | AKG(abcde) => SucCoA(0.5bcde,0.5edcb) + CO2(a) |
| v26 | SucCoA(abcd) <=> Suc(0.5abcd,0.5dcba) |
| v27 | Suc(0.5abcd,0.5dcba) <=> Fum(0.5abcd,0.5dcba) |
| v28 | Fum(0.5abcd,0.5dcba) <=> Mal(abcd) |
| v29 | Mal(abcd) <=> OAA(abcd) |
| v30 | Mal(abcd) => Pyr(abc) + CO2(d) |
| v31 | Ict(abcdef) => GOX(ab) + Suc(0.5fcde,0.5edcf) |
| v32 | GOX(ab) + AcCoA(cd) <=> Mal(abdc) |
| v33 | F6P => M6P |
| v34 | M6P => Alg |
| v35 | 2AcCoA => 3HB |
| v36 | 0.0228G3P + 0.0024PEP + 0.0012E4P + 0.0007G6P + 0.0194OAA + 0.2768AcCoA + 0.0062R5P + 0.0512Pyr + 0.0267AKG => Biomass |

**Supplemental Table 2**. Best estimates of net metabolic fluxes of *A.vinelandii* with 95% confidence intervals. Lb, lower bound; Ub, upper bound.

|  | wt without ammonium | | | AV3 without ammonium | | | wt with ammonium | | | AV3 with ammonium | | |
| --- | --- | --- | --- | --- | --- | --- | --- | --- | --- | --- | --- | --- |
| Reaction | Estimate | Lb | Ub | Estimate | Lb | Ub | Estimate | Lb | Ub | Estimate | Lb | Ub |
| v1 | 100.00 | 99.93 | 100.07 | 100.00 | 99.99 | 100.01 | 100.00 | 99.96 | 100.04 | 100.00 | 99.90 | 100.10 |
| v2 | 1.70 | 1.70 | 1.70 | 2.00 | 2.00 | 2.00 | 3.70 | 3.70 | 3.70 | 4.50 | 4.50 | 4.50 |
| v3 | 13.09 | -167.76 | 193.94 | 20.70 | -58.46 | 99.87 | 6.87 | -10.91 | 24.65 | 15.89 | -32.71 | 64.48 |
| v4 | 13.09 | -81.98 | 108.16 | 20.70 | 6.68 | 34.72 | 6.87 | 3.05 | 10.68 | 15.89 | 9.24 | 22.53 |
| v5 | 13.09 | -115.07 | 141.25 | 20.70 | -14.55 | 55.96 | 6.87 | 6.55 | 7.18 | 15.89 | -36.67 | 68.44 |
| v6 | 112.42 | -151.71 | 376.55 | 120.54 | 11.55 | 229.54 | 106.38 | 102.39 | 110.38 | 115.44 | 46.48 | 184.39 |
| v7 | 110.55 | 58.22 | 162.88 | 120.10 | 100.53 | 139.66 | 105.02 | 14.62 | 195.42 | 114.16 | -167.87 | 396.20 |
| v8 | 125.33 | 99.21 | 151.46 | 133.35 | 91.51 | 175.19 | 159.01 | 24.73 | 293.29 | 163.61 | 93.89 | 233.33 |
| v9 | 80.60 | 80.54 | 80.66 | 69.80 | 69.79 | 69.81 | 91.10 | 91.06 | 91.14 | 78.00 | 77.92 | 78.08 |
| v10 | 80.60 | 80.54 | 80.66 | 69.80 | 69.79 | 69.81 | 91.10 | 91.06 | 91.14 | 78.00 | 77.92 | 78.08 |
| v11 | 17.64 | 17.63 | 17.65 | 28.19 | 28.18 | 28.19 | 5.16 | 5.16 | 5.16 | 17.46 | 17.44 | 17.48 |
| v12 | 11.39 | -7.02 | 29.80 | 18.70 | 6.06 | 31.35 | 3.17 | -3.62 | 9.96 | 11.39 | -17.11 | 39.88 |
| v13 | 6.25 | -14.18 | 26.69 | 9.48 | -15.43 | 34.40 | 1.99 | 0.25 | 3.73 | 6.07 | -46.29 | 58.44 |
| v14 | 11.39 | -20.41 | 43.19 | 18.70 | 18.16 | 19.24 | 3.17 | -26.44 | 32.77 | 11.39 | 3.82 | 18.95 |
| v15 | -5.65 | -10.96 | -0.33 | -9.34 | -11.91 | -6.77 | -1.55 | -39.87 | 36.77 | -5.66 | -192.07 | 180.74 |
| v16 | -5.74 | -67.35 | 55.86 | -9.36 | -10.18 | -8.55 | -1.62 | -2.79 | -0.45 | -5.73 | -8.49 | -2.96 |
| v17 | -5.74 | -17.52 | 6.03 | -9.36 | -21.75 | 3.03 | -1.62 | -12.11 | 8.87 | -5.73 | -31.53 | 20.07 |
| v18 | 5.74 | -24.04 | 35.53 | 9.36 | -37.98 | 56.70 | 1.62 | -89.34 | 92.57 | 5.73 | -7.75 | 19.20 |
| v19 | 14.98 | -26.61 | 56.58 | 13.30 | -40.60 | 67.20 | 54.13 | -92.92 | 201.18 | 49.58 | -34.66 | 133.82 |
| v20 | 0.10 | -32.69 | 32.89 | 10.82 | -32.29 | 53.93 | 46.13 | -86.06 | 178.33 | 41.94 | -32.02 | 115.90 |
| v21 | 201.62 | 184.37 | 218.87 | 191.33 | 182.83 | 199.83 | 200.92 | 191.59 | 210.25 | 196.81 | 180.42 | 213.19 |
| v22 | 48.90 | 48.59 | 49.22 | 133.00 | 132.72 | 133.29 | 13.69 | 13.61 | 13.78 | 93.95 | 93.70 | 94.21 |
| v23 | 48.90 | 7.52 | 90.28 | 133.00 | -48.16 | 314.16 | 13.69 | 3.89 | 23.49 | 93.95 | -8.74 | 196.65 |
| v24 | 30.22 | 13.01 | 47.43 | 129.62 | 121.12 | 138.11 | 2.94 | -6.37 | 12.26 | 83.74 | 67.47 | 100.02 |
| v25 | 28.02 | 10.81 | 45.24 | 129.09 | 120.60 | 137.59 | 1.35 | -7.97 | 10.67 | 82.25 | 65.98 | 98.52 |
| v26 | 28.02 | -60.03 | 116.08 | 129.09 | 61.55 | 196.64 | 1.35 | -107.24 | 109.94 | 82.25 | -129.63 | 294.13 |
| v27 | 46.70 | 41.80 | 51.61 | 132.48 | 46.89 | 218.07 | 12.10 | 7.88 | 16.31 | 92.46 | 51.22 | 133.71 |
| v28 | 46.70 | -37.64 | 131.05 | 132.48 | 63.91 | 201.05 | 12.10 | -98.09 | 122.28 | 92.46 | -126.60 | 311.53 |
| v29 | 65.38 | -107.76 | 238.53 | 135.87 | 94.92 | 176.81 | 22.85 | -97.00 | 142.70 | 102.67 | -516.07 | 721.42 |
| ^a^v30 | 0 | 0 | 0 | 0 | 0 | 0 | 0 | 0 | 0 | 0 | 0 |  |
| v31 | 18.68 | 1.45 | 35.91 | 3.39 | -5.11 | 11.88 | 10.75 | 1.42 | 20.07 | 10.21 | -6.12 | 26.54 |
| v32 | 18.68 | -185.83 | 223.19 | 3.39 | -17.82 | 24.59 | 10.75 | -11.47 | 32.96 | 10.21 | -220.35 | 240.77 |
| ^b^v33 | 0 | 0 | 0 | 0 | 0 | 0 | 0 | 0 | 0 | 0 | 0 | 0 |
| ^b^v34 | 0 | 0 | 0 | 0 | 0 | 0 | 0 | 0 | 0 | 0 | 0 | 0 |
| v35 | 55.63 | 55.49 | 55.78 | 24.76 | 24.62 | 24.90 | 79.97 | 79.96 | 79.99 | 38.59 | 38.50 | 38.68 |

The cells were harvested at 46 hr for wt and AV without added ammonium, and AV3 with added ammonium, and 27 hr for wt with added ammonium for flux estimation, corresponding to specific growth rates at these time points of 0.022 ± 0.009, 0.016 ± 0.009, 0.043 ± 0.011, 0.025 ± 0.007, for wt and AV3 without added ammonium, and wt and AV3 with added ammonium, respectively. ^a^ Flux from malate to pyruvate was also set to zero because the measured flux ratio of pyruvate from malate is negligible. ^b^ Fluxes through alginate formation were set to zero as no alginate was detected in any condition.

(A)


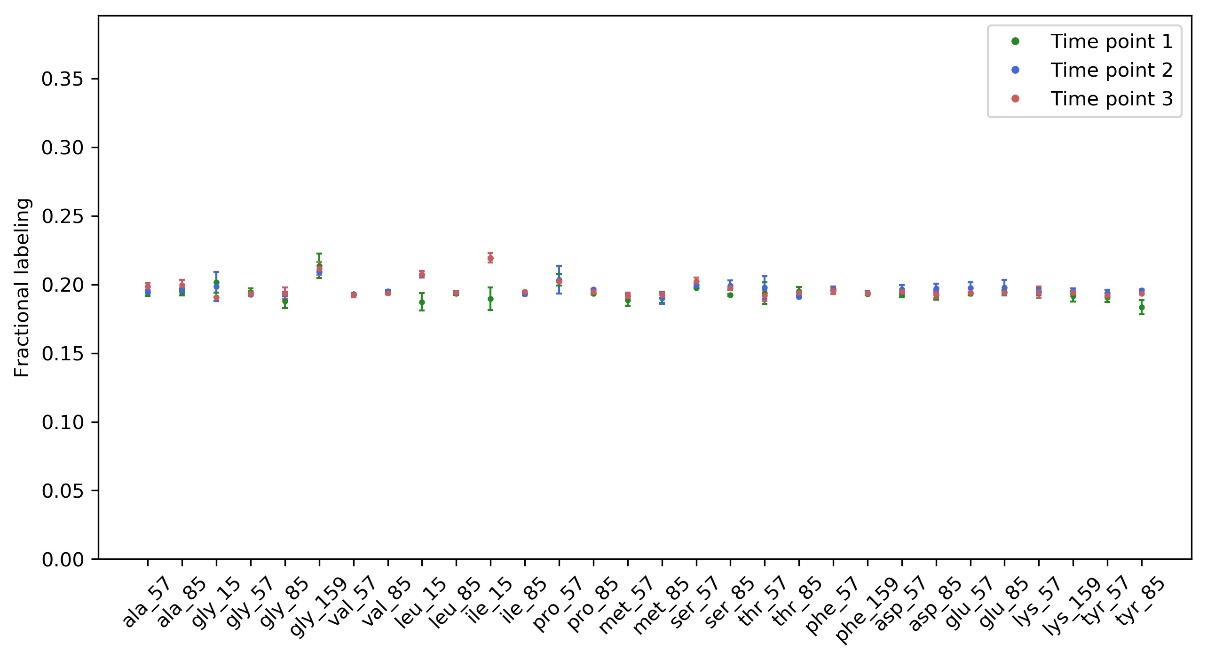


(B)


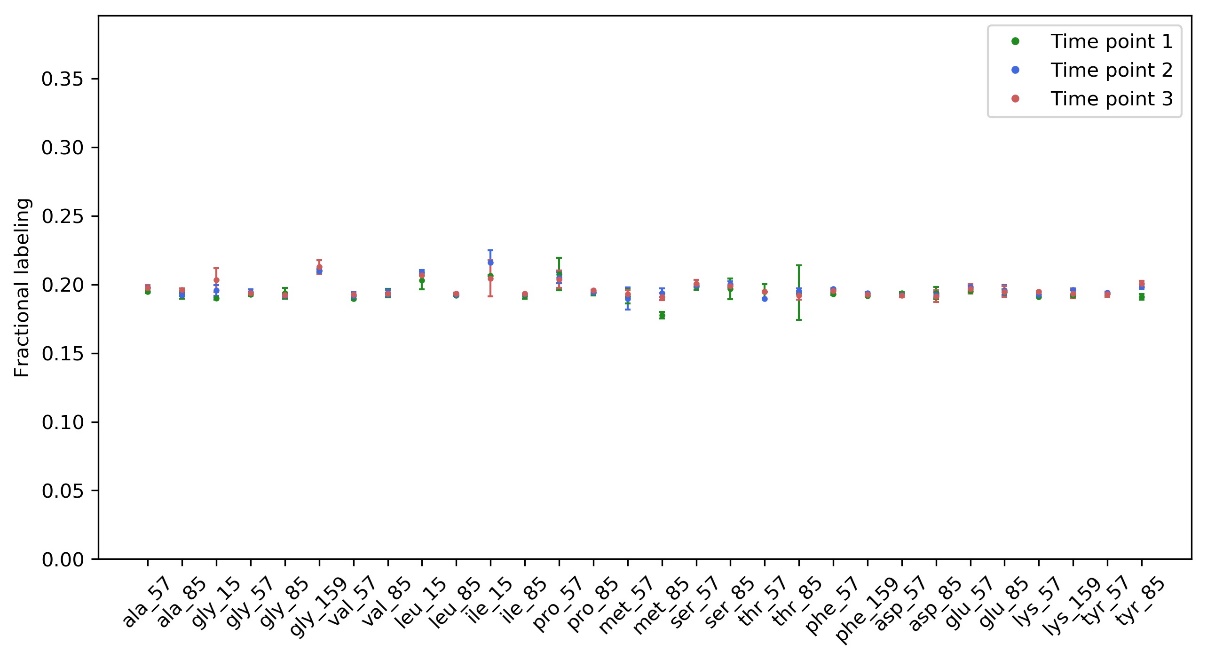


(C)


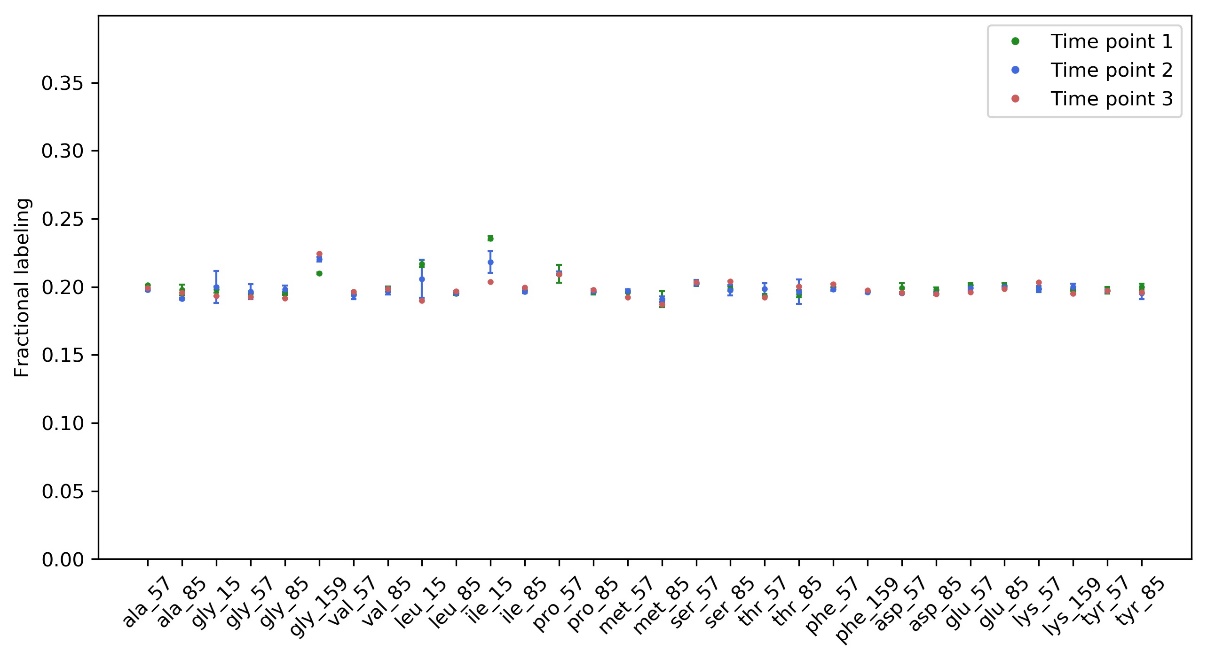


(D)


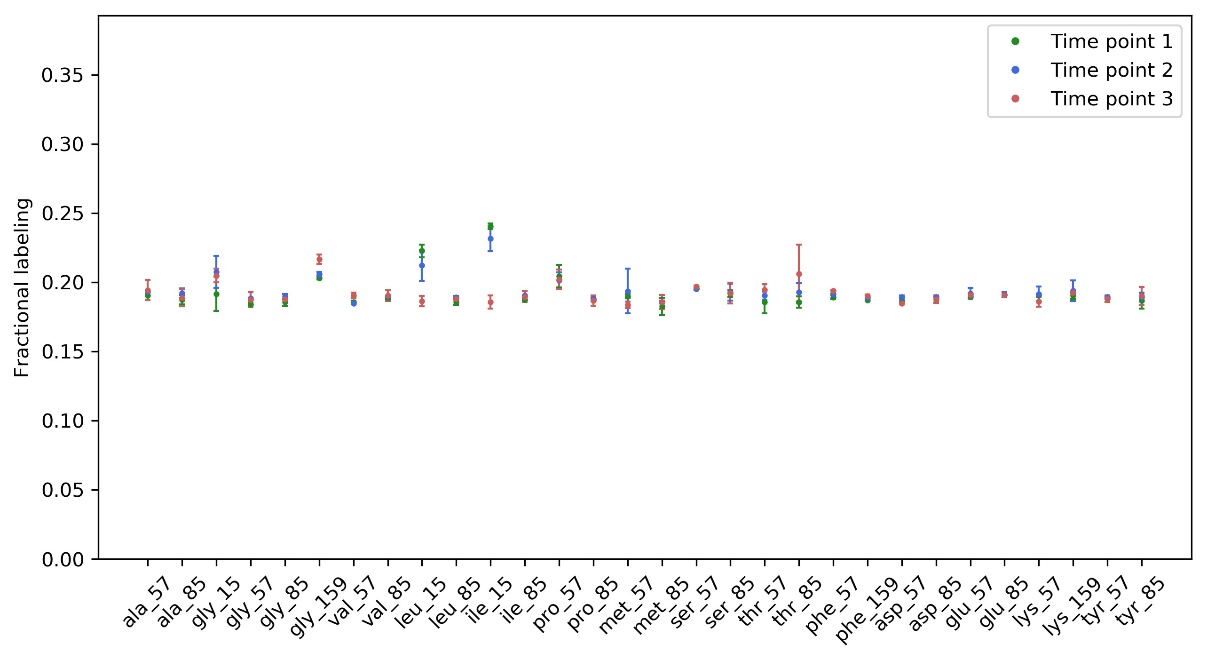


**Supplemental Figure 1.**

Metabolic steady state of *A.vinelandii* during metabolic flux analysis. Samples were taken at three different time points (as described in the main manuscript; 42, 46 and 50 hr for wt and AV3 without added ammonium, and AV3 with added ammonium; 25, 27 and 29 hr for wt with added ammonium) during the growth of (A) wt without additional ammonium, (B) AV3 without additional ammonium, (C) wt with added 50 mM ammonium and (D) AV3 with added 50 mM ammonium. Cells were cultivated with a mixture of 20% U-^13^C glucose and 80% unlabeled glucose. Fractional labeling of amino acids were calculated from their MDVs.


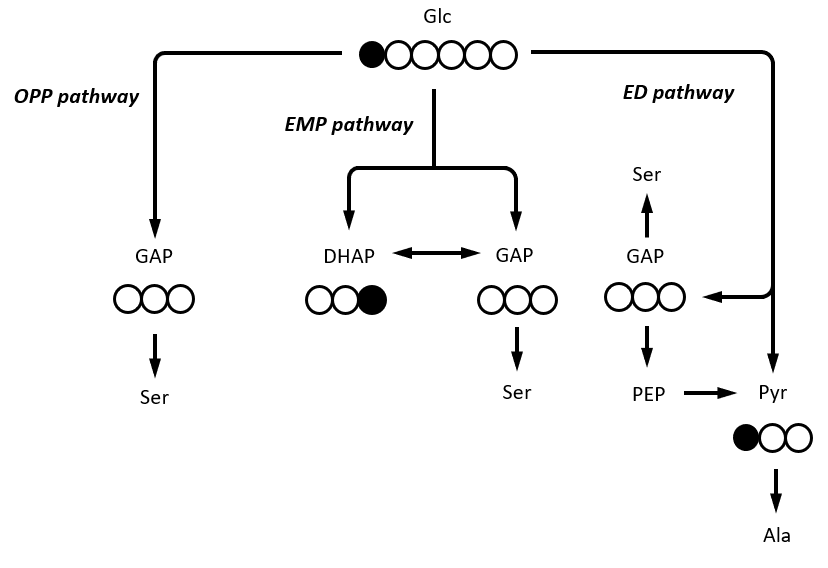


**Supplemental Figure 2.**

Labeling patterns of intermediates in glycolytic pathways using 1-^13^C glucose. GAP can derive through the EMP pathway, OPP pathway and ED pathway. Ideally, if GAP originates from the EMP pathway, half of the serine molecules will be labeled at position 3 while the other half will be unlabeled. If all GAP originates from the OPP pathway, none of the molecules will be labeled due to the loss of C1 in the oxidative phase. The ED pathway also produces an unlabeled GAP, and a C1 labeled Pyr. Abbreviations: Ala, alanine; DHAP, dihydroxyacetone phosphate; GAP, glyceraldehyde-3-phosphate; Glc, glucose; Pyr, pyruvate; Ser, serine; EMP pathway, Embden-Meyerhof-Parnas pathway; ED pathway, Entner-Doudoroff pathway; OPP pathway, oxidative pentose phosphate pathway.

(A)


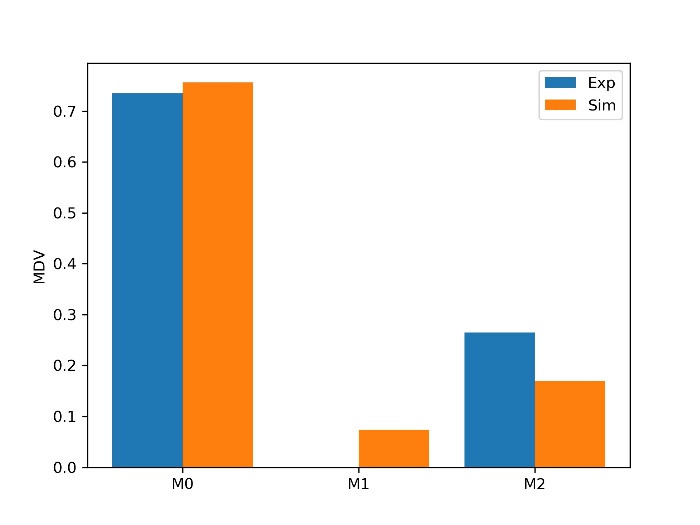

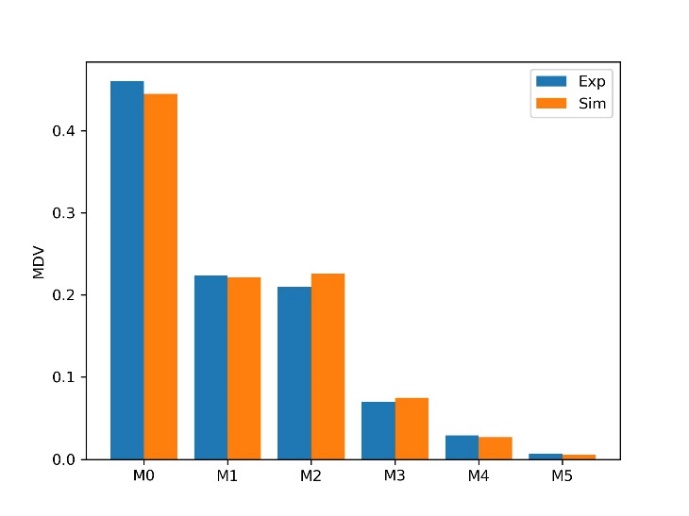


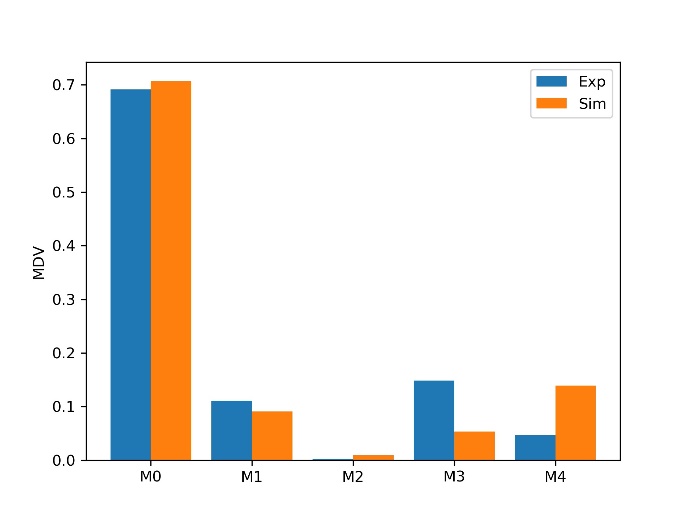

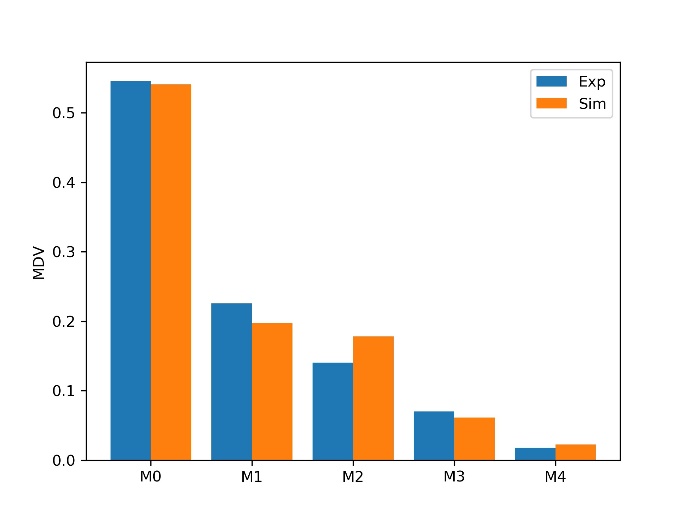


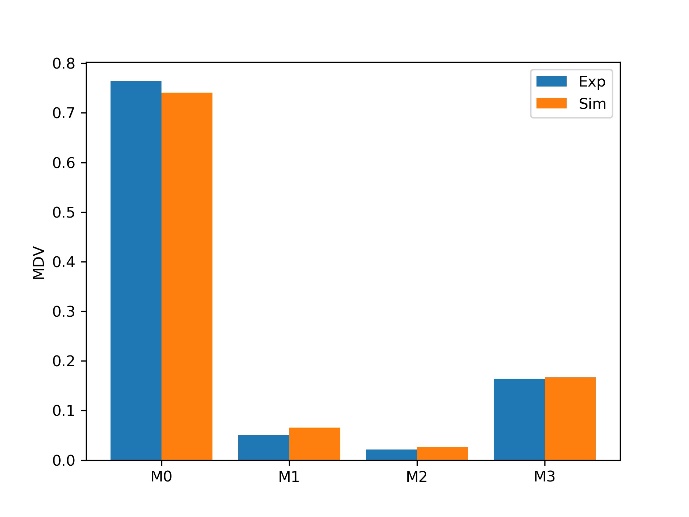

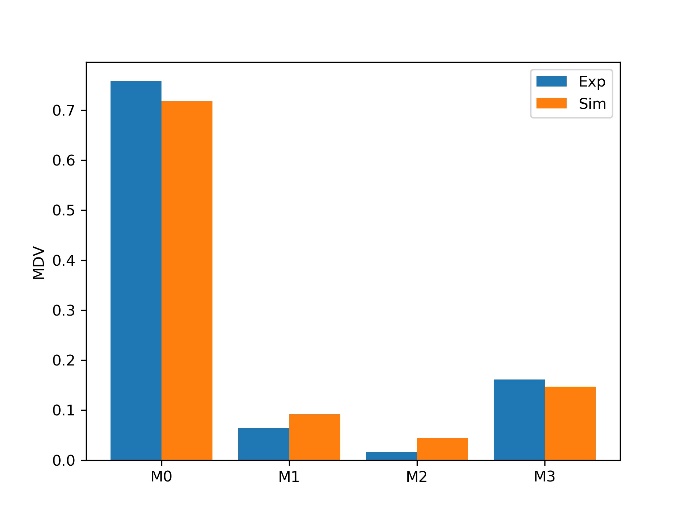


(B)


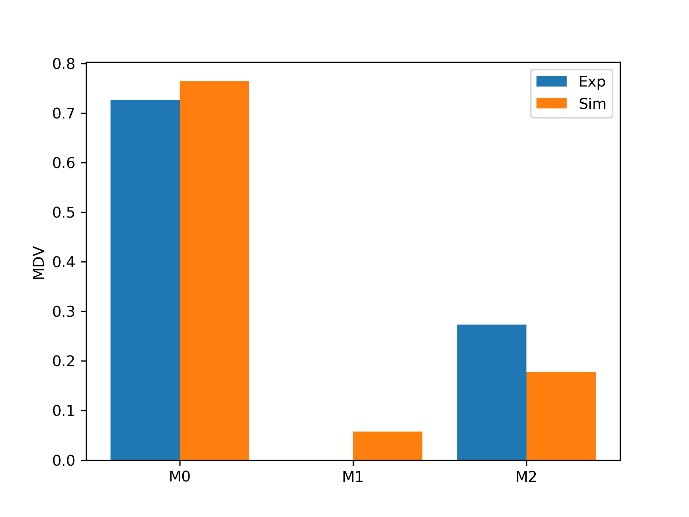

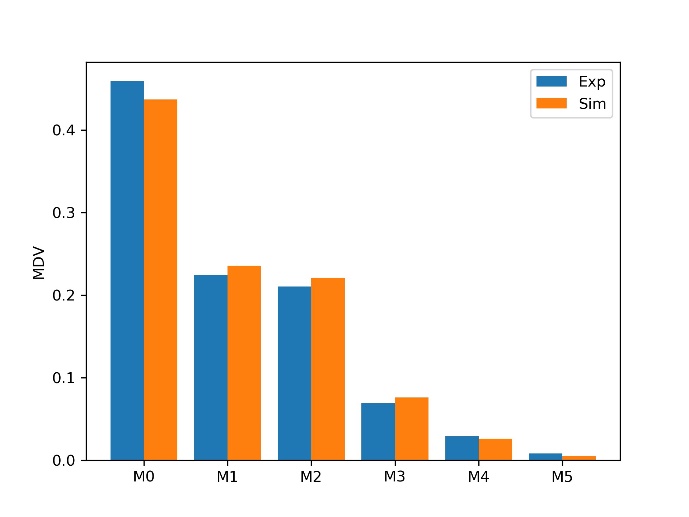


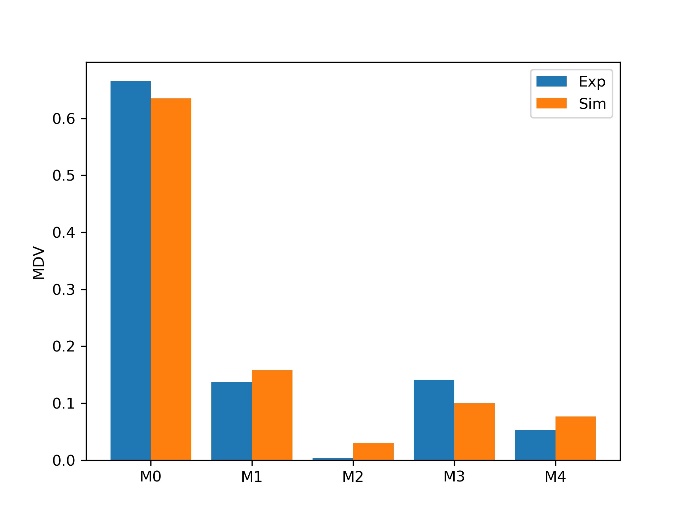

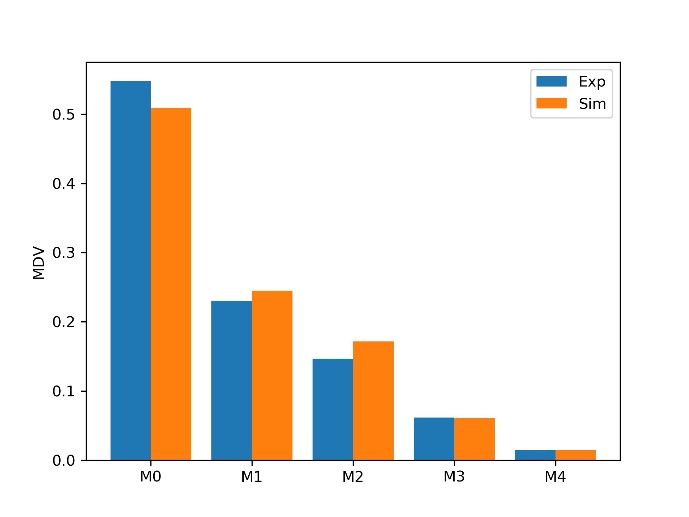


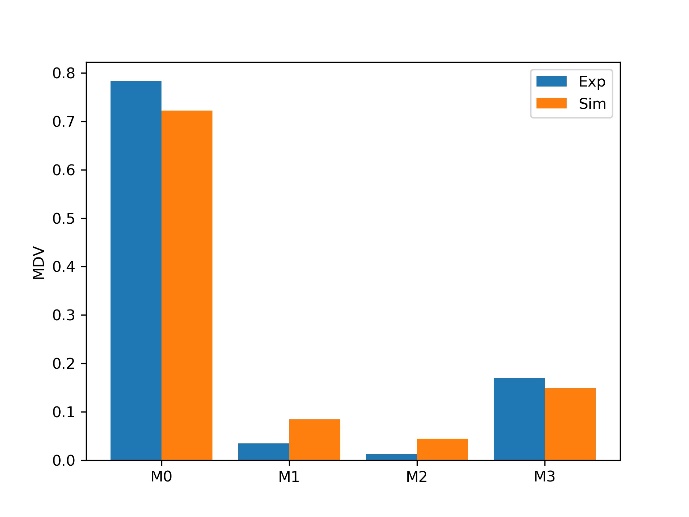

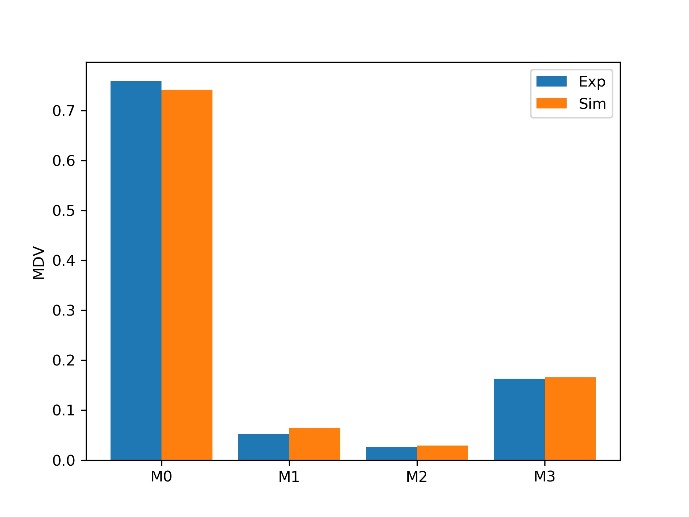


(C)


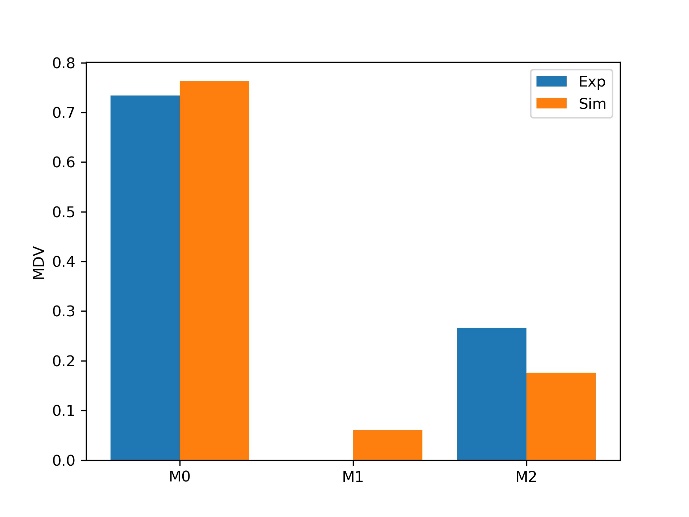

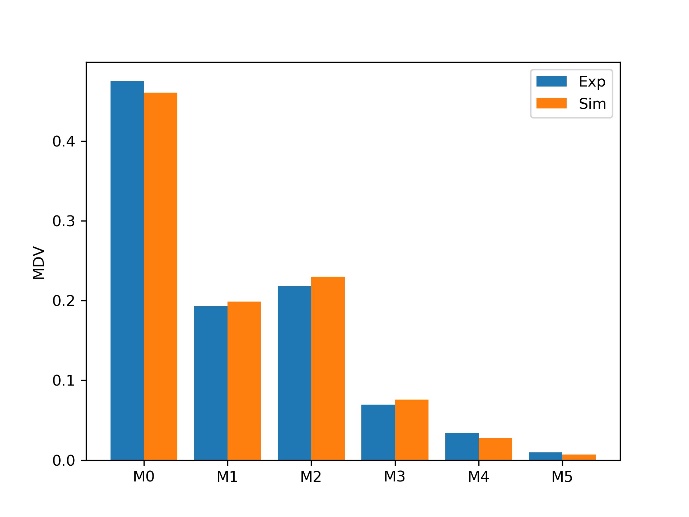


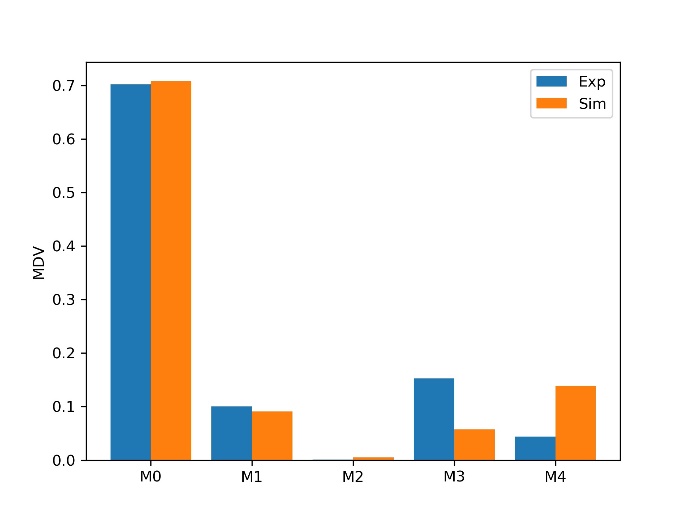

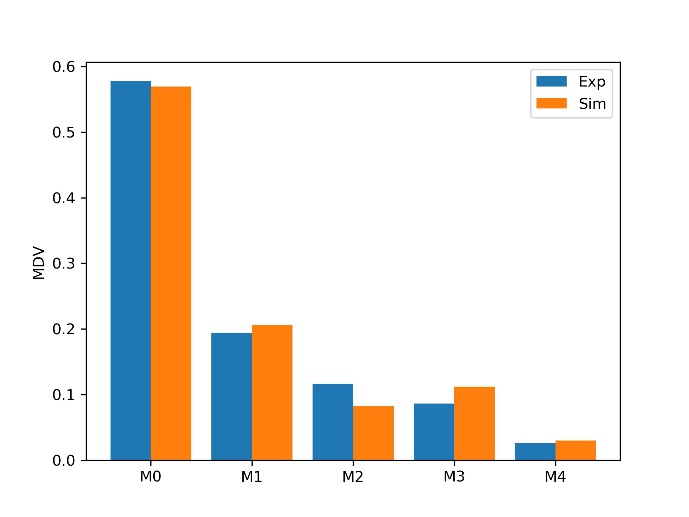


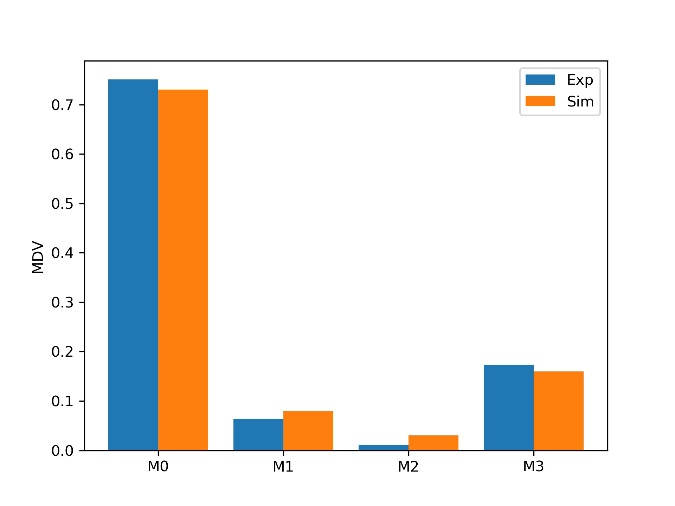

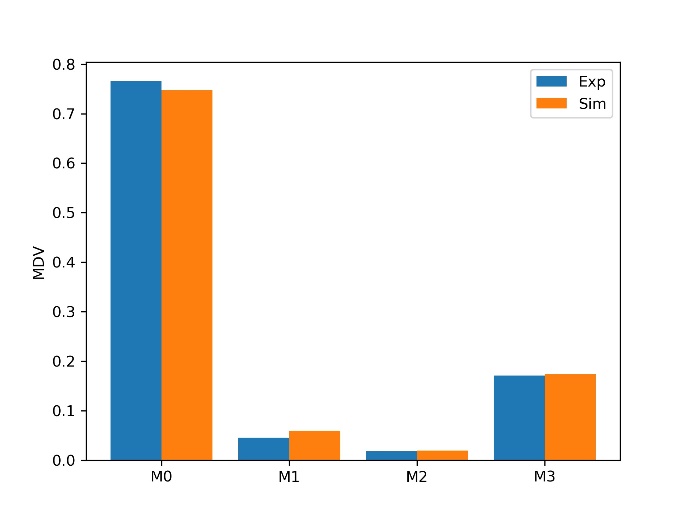


(D)


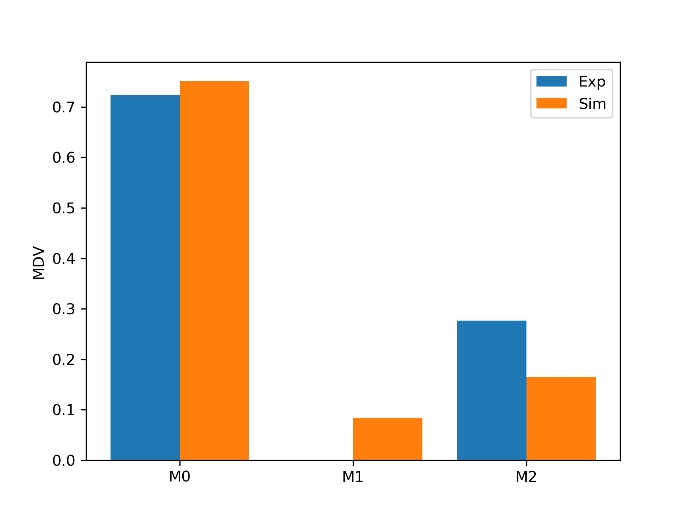

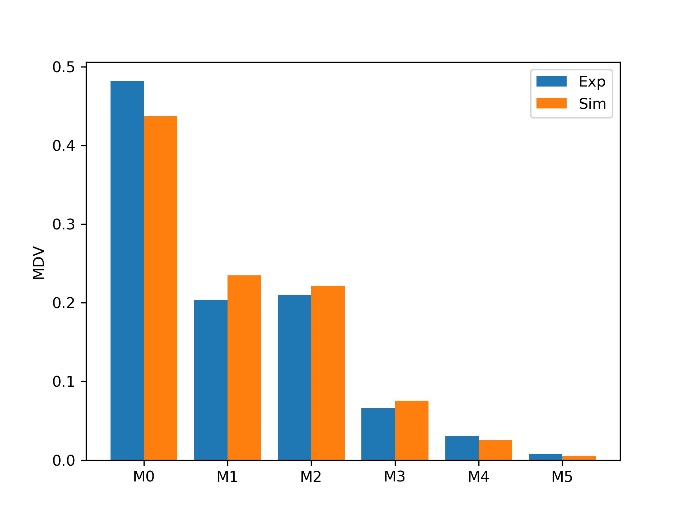


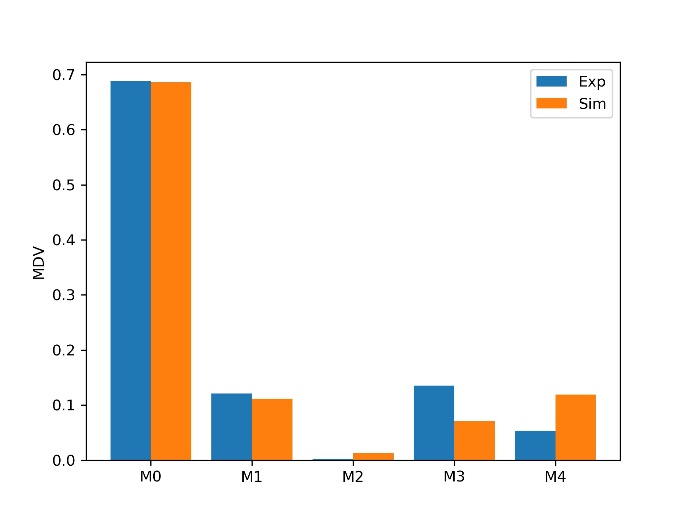

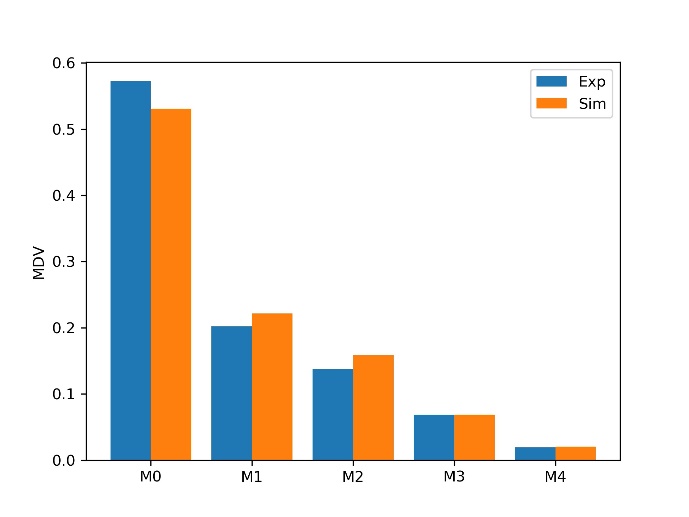


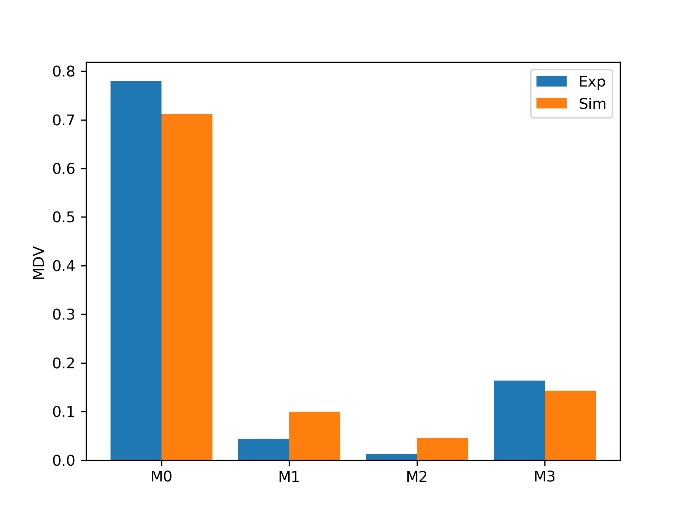

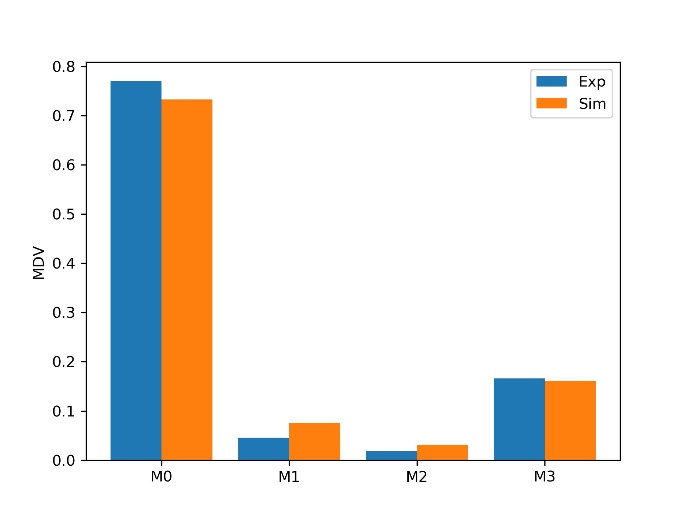


**Supplemental Figure 3.**

Metabolic flux model estimation fitting results of *A.vinelandii*. Experimental (blue) bar and simulated (orange) bar representing MDV of AcCoA, AKG, E4P, OAA, PEP, and Pyr are plotted from left to right and from top to bottom for (A) wt without additional ammonium, (B) AV3 without additional ammonium, (C) wt with added 50 mM ammonium and (D) AV3 with added 50 mM ammonium. Abbreviations: AcCoA, acetyl coenzyme A; AKG, a-ketoglutarate; E4P, erythrose-4-phosphate; OAA, oxaloacetate; PEP, phosphoenolpyruvate; Pyr, pyruvate.


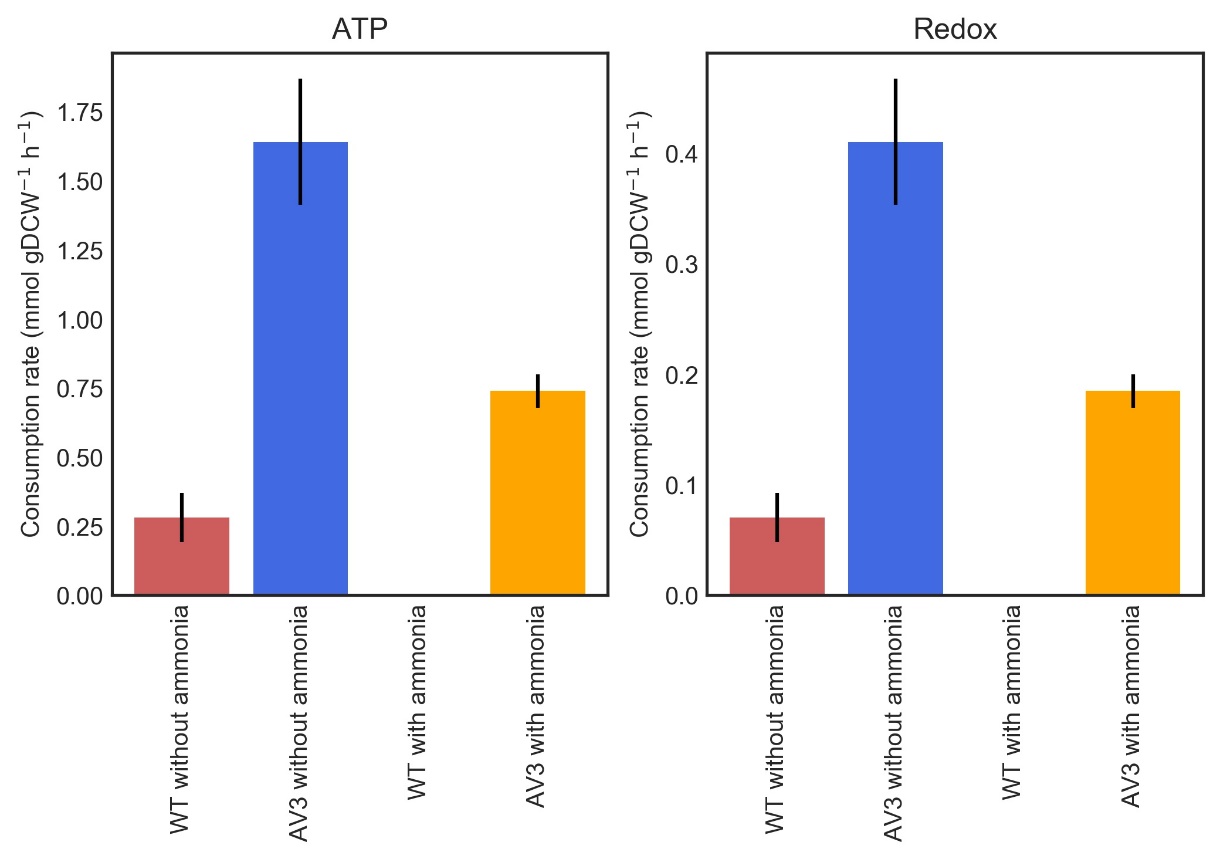


**Supplemental Figure 4.**

Energy and reducing equivalent consumption in nitrogen fixation of *A. vinelandii* wt and AV3 with and without ammonium. ATP and redox consumption rates were obtained from estimated fluxes, measured extracellular ammonium and stoichiometry of corresponding reactions. ATP and redox utilization of the wt strain with added ammonium were zero because the nitrogen requirement for biomass was calculated to be fully derived from supplied ammonium, whereas the AV3 mutant with supplied ammonium maintains an extracellular concentration of ammonium, consistent with some nitrogenase activity.
